# Harnessing immunotherapy: cancer vaccines as novel therapeutic strategies for brain tumor

**DOI:** 10.3389/fimmu.2025.1588081

**Published:** 2025-07-17

**Authors:** Klaudia Kiel, Raziye Piranlioglu, Jakub Godlewski, Agnieszka Bronisz

**Affiliations:** ^1^ Tumor Microenvironment Laboratory, Mossakowski Medical Research Institute, Polish Academy of Sciences, Warsaw, Poland; ^2^ Department of Neurosurgery, Brigham and Women’s Hospital, Harvard Medical School, Boston, MA, United States; ^3^ Department of Neurooncology, Mossakowski Medical Research Institute, Polish Academy of Sciences, Warsaw, Poland

**Keywords:** vaccine, immunotherapy, glioblastoma, cancer vaccine, brain tumor

## Abstract

Cancer vaccines have emerged as a pivotal area of research in oncology, demonstrating significant promise in harnessing the immune system to combat cancer. Recent advancements in antigen identification and sequencing techniques have catalyzed the development of cancer vaccines whose goal is to elicit robust humoral and cellular immune responses against cancer cells. Despite their potential, most cancer vaccines are still in the experimental phase, primarily due to challenges associated with tumor-induced immune suppression. This article explores the role of cancer vaccines in brain cancer, glioblastoma, by providing a granular analysis of clinical trial results and mechanisms of resistance alongside a comparative assessment. These vaccines aim to navigate the immunosuppressive tumor microenvironment by targeting glioblastoma-specific antigens, offering new hope for improved treatment outcomes. The unique mechanisms defining cancer vaccines, such as their ability to activate dendritic cells and T cells, underscore their precision in selectively attacking cancer cells while sparing healthy tissue. Furthermore, the categorization of these vaccines into preventive and therapeutic types, along with various delivery methods, illustrates their diverse capacity. Finally, this review highlights the potential impact of cancer vaccine clinical trials on future cancer therapies, where effective anti-cancer strategies are within reach. It also provides an in-depth discussion of the brain tumor microenvironment and its influence on vaccine efficacy.

## 1 Introduction of cancer vaccines

### 1.1 Definition and mechanisms

Cancer vaccines, also known as cancer antigen vaccines, are at the forefront of oncological innovation, driven by rapid advancements in medical science. These vaccines are engineered to harness tumor-specific antigens, activating the body’s immune defenses to target cancer cells precisely while sparing healthy tissue. They work by introducing these antigens into dendritic cells (DCs), which stimulate T cells to attack cancer, akin to how viral vaccines train the immune system to target virus-infected cells (1–11). This method is particularly effective against non-solid tumors, such as hematological malignancies, which are more accessible to immune interventions and reside in less immunosuppressive environments than solid tumors (12).

Cancer vaccines are engineered to activate both cellular and humoral immune responses against tumor-specific antigens. However, due to the profound immunosuppressive environment orchestrated by brain tumors, cancer vaccines alone are insufficient to reverse immune suppression. Consequently, integrating vaccines with immune-modulating agents, such as checkpoint inhibitors or cytokine therapies, may be crucial for achieving optimal therapeutic efficacy (13).

Despite their potential, cancer vaccines face substantial hurdles, mainly when targeting brain tumors. The brain’s unique challenges, such as the blood-brain barrier (BBB) and its immune-privileged status, complicate vaccine delivery and efficacy. The dense, fibrous stroma of the brain and the immunosuppressive microenvironment of tumors, such as glioblastoma, further impede immune activity against these malignancies. As a result, while effective in more immunologically active tumors, the application of cancer vaccines in brain tumors demands innovative strategies to overcome these obstacles (14).

**Figure 1:**
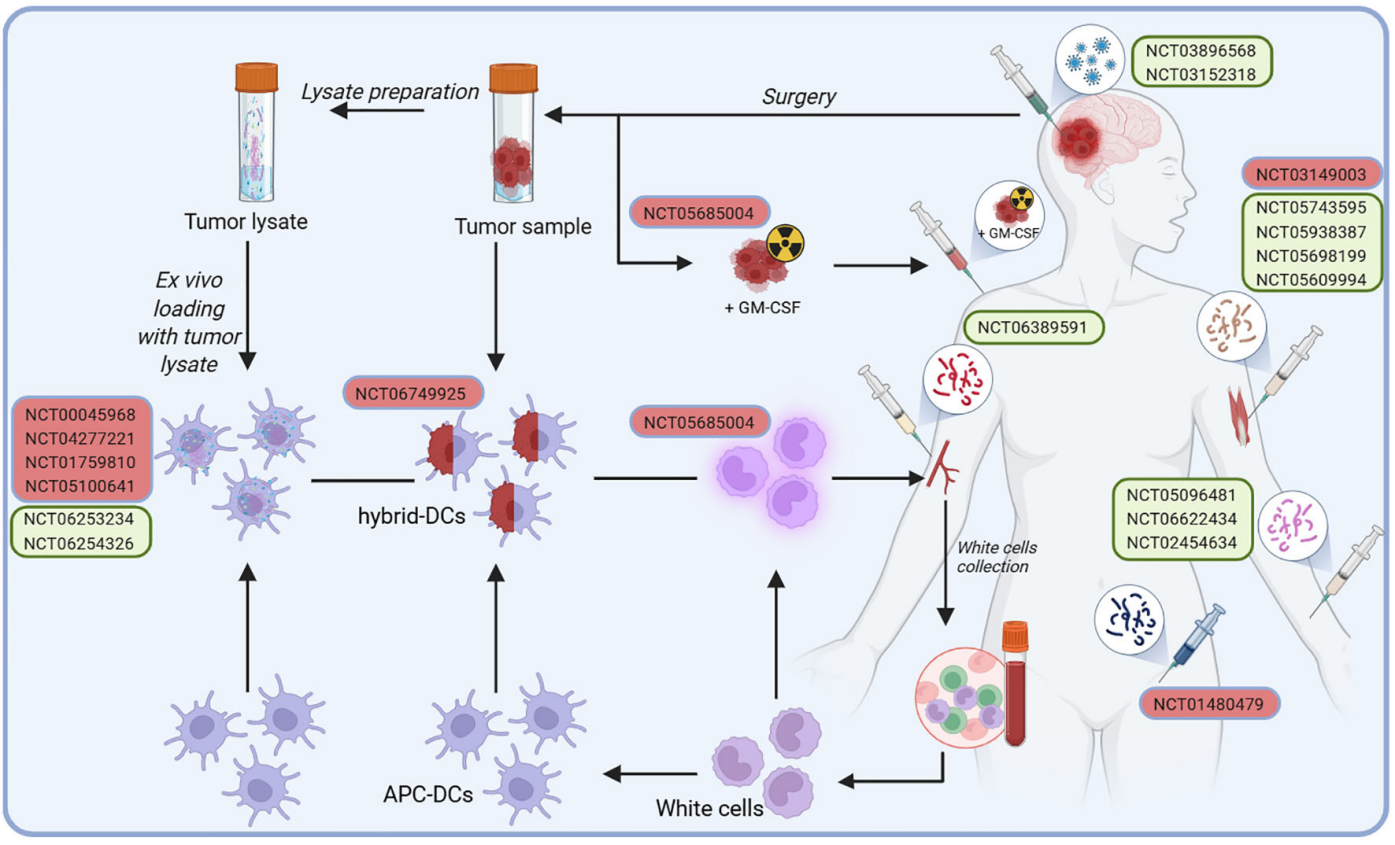
Developing cancer vaccines for glioblastoma patients in clinical trials follows a structured process designed to stimulate an immune response. In addition to conventional administration routes, such as intramuscular, subcutaneous, or injection into the post-surgical tumor cavity, some approaches involve a more complex preparation process. It begins with harvesting a tumor sample during surgery, from which cancer cells are isolated or processed into a tumor lysate rich in tumor-specific antigens. Simultaneously, white blood cells are collected from the patient and differentiated into antigen-presenting cells (APCs), specifically DCs. Tumor antigens are thus either loaded onto DCs or fused with them to create hybrid DCs capable of effectively presenting tumor-associated antigens ex vivo. Once activated, these DCs are reintroduced into the patient as a vaccine, aiming to trigger a robust immune response against the tumor.

The structured development process of cancer vaccines used in clinical trials for glioblastoma patients is illustrated in **Figure 1**. Initially, a tumor sample is harvested during surgical resection, from which tumor cells are isolated or processed into tumor lysates enriched with tumor-specific antigens. Concurrently, patient-derived white blood cells are collected and differentiated into antigen-presenting cells (APCs), particularly DCs. Tumor antigens are then loaded onto or fused with DCs, creating antigen-presenting DCs or hybrid DCs capable of effectively presenting tumor-associated antigens ex vivo. These prepared DC vaccines are reintroduced into the patient, utilizing various administration routes, including intramuscular, subcutaneous, or direct injection into the post-surgical tumor cavity, to stimulate a targeted immune response against glioblastoma.

Cancer vaccines continue to hold promise as research progresses to revolutionize cancer therapy. These vaccines aim to mirror the impact of traditional antimicrobial vaccines on global health, offering hope for new treatments for previously intractable cancers and suggesting a future where cancer might be managed or even prevented through immunological strategies (15–24).

**Figure 2:**
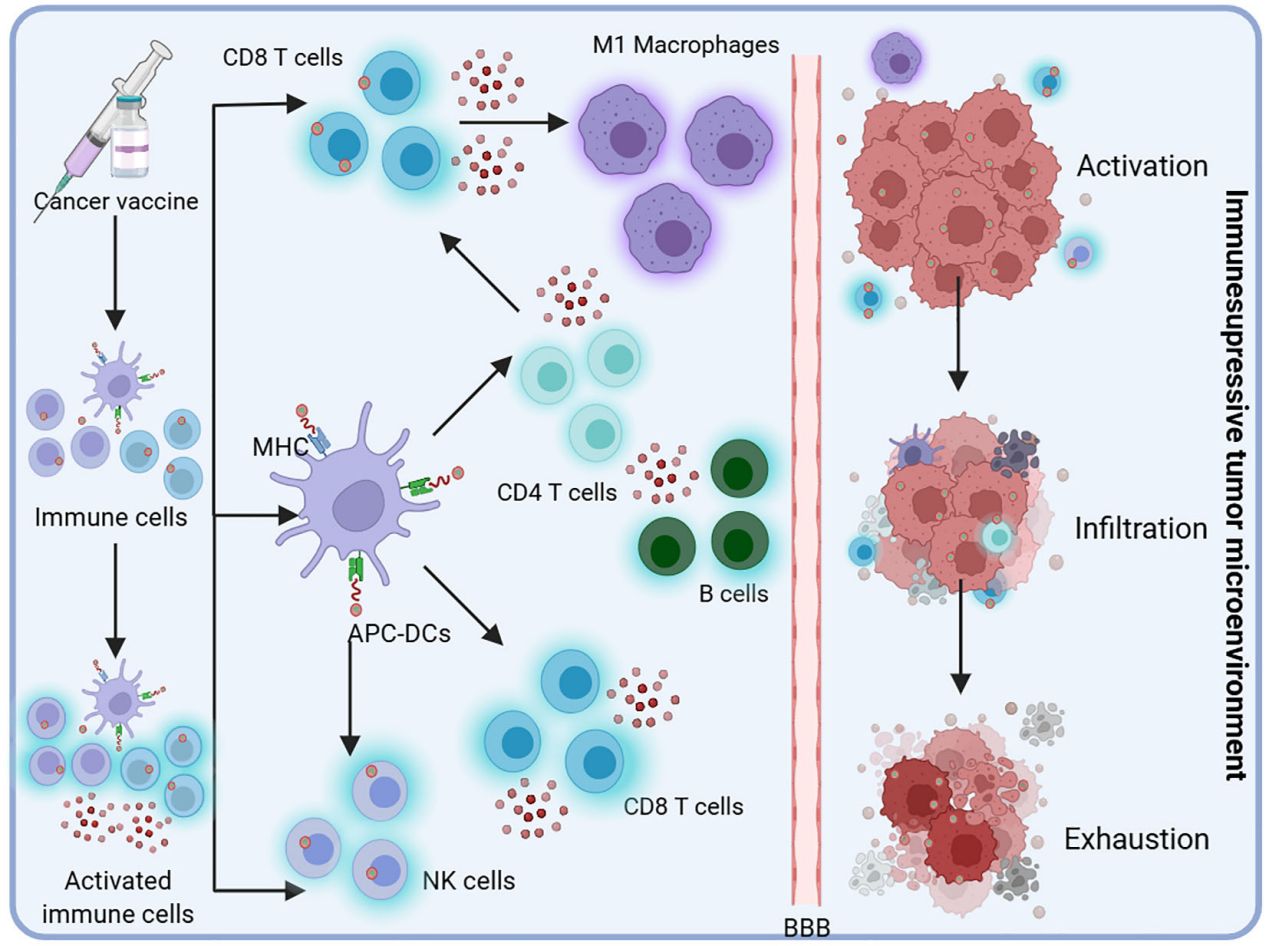
Upon administration, the vaccine initiates an immune cascade. APCs, such as DCs, recognize and process the introduced tumor antigens at the site of injection. These cells present the processed antigens on their Major Histocompatibility Complex (MHC) molecules to immune effectors, including natural killer (NK) cells and CD4+ and CD8+ T cells. The activated T cells proliferate and secrete cytokines, thereby amplifying the immune response by recruiting additional immune cells, such as M1 macrophages and B cells. These activated immune cells circulate throughout the body, seeking out and attacking tumor cells. However, glioblastoma presents significant challenges, such as the BBB and an immunosuppressive tumor microenvironment, which limit immune cell infiltration and function, ultimately contributing to immune evasion and continued tumor growth.

The immune cascade initiated upon vaccine administration is depicted in **Figure 2**. At the injection site, APCs such as DCs recognize and process the introduced tumor antigens, presenting them on their Major Histocompatibility Complex (MHC) molecules to immune effector cells, including natural killer (NK) cells and CD4+ and CD8+ T cells. Activated T cells proliferate and release cytokines, amplifying the immune response by recruiting additional immune cells such as M1 macrophages and B cells. These activated immune cells circulate through the whole body, seeking out and attacking tumor cells. Despite the robust immune activation, significant impediments such as the BBB and the immunosuppressive tumor microenvironment (TME) characteristic of glioblastoma present substantial challenges, restricting immune cell infiltration and functionality and consequently facilitating immune evasion and tumor persistence.

### 1.2 Historical development

The concept of leveraging the immune system to combat cancer has deep historical roots, tracing back to the late 19th century. This notion was initially explored through pioneering treatments, such as “Coley’s toxins,” a concoction of bacterial products used by William Coley to induce an immune response against tumors. Although the results were, at best, inconsistent (25), they set the stage for a century of innovation in cancer immunotherapy.

Throughout the 20th century, significant strides were made in understanding and harnessing the immune system’s capabilities against cancer. A key milestone in the 1950s was the discovery of interferons, proteins that play a critical role in defense against pathogens. Their immune-modulating effects have made them a cornerstone in the treatment of various cancers since (26–30).

The latter half of the century saw the development of the first cancer vaccines targeting specific antigens found on the surface of cancer cells. These vaccines aim to present these cancer antigens to the immune system, training it to recognize and destroy cancer cells. The Bacillus Calmette–Guérin (BCG) vaccine, approved for bladder cancer in 1990, stands out as a landmark example, showcasing the potential of directly stimulating the immune system to combat cancer (31).

The advent of genetic engineering further revolutionized cancer immunotherapy, and the development of chimeric antigen receptor (CAR) T-cell therapy, a technique that genetically modifies a patient’s T cells to target and attack cancer cells, marked a significant breakthrough. This innovation, which demonstrated profound success, especially in treating certain types of leukemia and lymphoma, highlighted the potential for engineered immune cells in oncology (32).

Recent decades have witnessed transformative advancements with the introduction of mRNA vaccine technologies. Using synthetic mRNA encoding tumor antigens, these vaccines represent a significant leap forward in precision medicine. The patient’s cells produce the tumor antigen by delivering this mRNA into the body, eliciting a robust immune response. This technology gained global attention during the COVID-19 pandemic but has been explored in oncology for years, particularly for its potential to tailor treatments to the genetic makeup of individual tumors (16).

In the case of glioblastoma, a particularly aggressive and challenging brain cancer, immunotherapy has been slow to yield consistent results. However, the development of vaccines targeting glioblastoma antigens, such as the Epidermal Growth Factor Receptor Variant III (EGFRvIII) peptide, offers hope for enhancing immune responses against these tumors. Clinical trials using vaccines like ImuVert and oncolytic viruses (OV) have shown promising survival benefits, but challenges remain in overcoming the tumor’s ability to evade immune detection (33–35). Research efforts continue to refine vaccine strategies, incorporating novel approaches such as DC vaccines and immune checkpoint inhibitors to enhance therapeutic efficacy.

**Figure 3:**
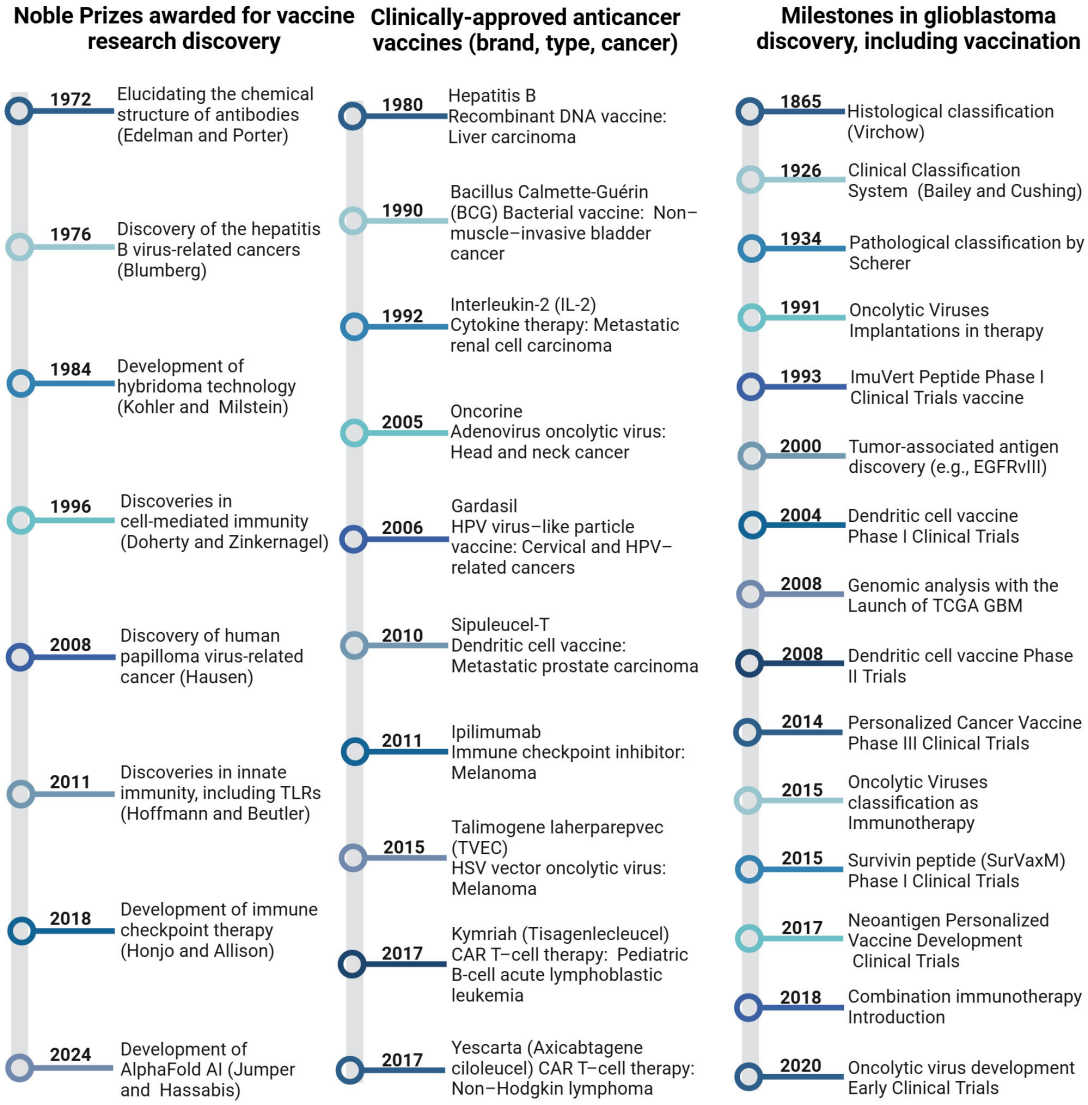
Timelines of milestones in cancer immunotherapy, including glioblastoma, provide a chronological overview of significant developments and achievements.

Ongoing research into glioblastoma vaccines continues to explore ways to improve the immune system’s ability to recognize and attack these cancer cells, offering potential new avenues for treatment. The concurrent timelines below highlight key milestones in glioblastoma research and vaccine development, illustrating the progression of immunotherapeutic strategies from the late 19th century to the present (**Figure 3**) (9, 21, 25, 30, 34–50).

## 2 Types of cancer vaccines

### 2.1 Preventive cancer vaccines

Preventive cancer vaccines aimed at reducing cancer incidence by targeting specific pro-oncogenic viruses represent a significant advancement in oncology. Among the most prominent and well-documented success stories is the human papillomavirus (HPV) vaccine, which effectively prevents cervical cancer by neutralizing the HPV responsible for most cervical cancer cases (51). Similarly, Hepatitis B (HBV) vaccines have drastically reduced liver cancer rates by preventing HBV infections that can progress to hepatocellular carcinoma (52–54). Despite these achievements, the application of preventive vaccines in central nervous system (CNS) malignancies remains elusive. For example, while cytomegalovirus (CMV) appears to be prevalent in glioblastoma cells, a direct oncogenic link has not been established, which restricts the potential for virus-targeted preventive strategies in these cancers. Still, clinical trials targeting CMV antigens, including peptide and RNA vaccines, are ongoing (22 clinical trials for glioblastoma (20/01/25), with some showing potential for inducing robust immune responses (55, 56). However, the complexity of brain tumors, including their origins and the BBB, poses unique challenges that prevent straightforward applications of preventive vaccines developed for other cancers. Given these challenges, research has shifted toward more tailored preventive strategies that might benefit individuals at high genetic risk of developing brain tumors. For instance, individuals with genetic conditions like Li-Fraumeni syndrome or Turcot syndrome, which significantly increase the risk of developing various cancers, including brain tumors, could potentially benefit from vaccines targeting specific tumor antigens that arise during early oncogenic processes (57, 58). These vaccines aim to train the immune system to recognize and eliminate cells exhibiting early signs of malignant transformation before they can develop into full-blown tumors. Current research on preventive strategies for brain tumors includes early-phase clinical trials that explore vaccines targeting unique or overexpressed antigens specific to cancer development in the CNS (59, 60). These trials will assess the viability of preventive vaccines in a field where such interventions are not yet established. In conclusion, due to the absence of specific symptoms and biomarkers, preventing primary brain tumors remains an unresolved challenge. Prevention efforts are thus primarily focused on therapies aimed at preventing recurrence instead.

### 2.2 Therapeutic cancer vaccines

While preventive vaccines have shown considerable success in combating cancers linked to viral infections, such as cervical and liver cancers, their role in CNS malignancies remains limited due to the lack of confirmed viral origins for these tumors. The focus in neuro-oncology has thus shifted towards therapeutic vaccines, which promise to improve outcomes by explicitly targeting the unique molecular features of brain tumors. Unlike preventive vaccines, therapeutic vaccines for CNS malignancies are intended to treat existing tumors. These vaccines aim to enhance the immune system’s ability to recognize and destroy cells that express specific tumor antigens. In glioblastoma, for example, vaccines targeting the EGFRvIII mutation—a variant uniquely found in a sizeable sub-population of glioblastoma patients—have been developed and are undergoing clinical trials (4). These therapeutic vaccines are designed to induce an immune response specifically against tumor cells harboring this mutation, illustrating a targeted approach not typically feasible with preventive vaccines. The progress in the development of therapeutic vaccines is underscored by clinical trials such as those testing DC vaccines like DCVax-L, which involve loading DCs with antigens from a patient’s tumor, thereby instructing the immune system to recognize and attack glioblastoma cells. However, these trials, advancing through phases 2 and 3, have sparked controversy in the neuro-oncology field due to flaws in their study design, with many raising concerns about using external and historical controls, as well as the definition of trial endpoints (2, 61–63).

The field continues to innovate with genomic technologies aiding the development of personalized vaccines. These vaccines are tailored to the unique antigenic profiles of each patient’s tumor, potentially enhancing the specificity and effectiveness of the immune response in CNS malignancies.

## 3 Understanding brain tumor complexity

### 3.1 Overview of brain tumor glioblastoma

Glioblastoma is a highly aggressive and the most common primary brain tumor that disproportionately affects older individuals. In 1926, Percival Bailey and Harvey Cushing, during the pioneering analysis of brain tumors, described undifferentiated glial precursors with variable phenotypic traits within necrotic and hemorrhagic tissue, coining the now-historical term *glioblastoma multiforme* (37). Since then, their findings have been corroborated by transcriptional profiling of the bulk tumor (TCGA GBM, 2008), *in situ* gene expression (Ivy Gap, 2016), and single-cell deep sequencing (Single Cell Portal, 2014). Revealed by those genomic sequencing approaches, genetic alterations in various genes, such as TP53, EGFR (EGFRvIII mutation), and PDGFR, or hypermethylation of CpG promoters (O-6-Methylguanine-DNA Methyltransferase (MGMT) promoter methylation is a key predictor of response to temozolomide in genes related to tumor formation and progression (64). While these alterations contribute to the aggressive nature of glioblastoma and therapeutic response, they provide crucial tumor-specific markers that enable therapeutic intervention and diagnostics (65–67). Nevertheless, the effectiveness of experimental gene therapies is still patchy (68, 69). The primary reason is the pervasive heterogeneity perceived almost a century ago, which is manifested by the coexistence of diverse cell subtypes within most patients’ tumors, thereby blunting the effectiveness of most targeted therapeutic approaches.

Glioblastoma has an abysmal median survival rate of 15 months from the time of diagnosis, resulting in a very short therapeutic window (70). Such a dismal prognosis is, in addition to the tumor’s high cellular heterogeneity and aggressiveness, further confounded by infiltrative growth patterns, interactions with the CNS, and resistance to treatment. Glioblastoma stimulates angiogenesis, remodels the extracellular matrix, and contains a significant admixture of glioblastoma stem-like cells (GSCs) that are resistant to standard therapies, leading to tumor recurrence. Additionally, the sheer existence of a BBB and an immune-suppressive tumor microenvironment (TME) further thwarts therapeutic interventions for glioblastoma (70–72).

### 3.2 Brain tumor immunological barriers

The glioblastoma TME is marked by an abundance of immune-suppressing regulatory T cells (Tregs), myeloid-derived suppressor cells (MDSCs), and a deficiency of T cells, further compounded by the BBB, which limits the infiltration of immune cells and therapeutic/drugs into the TME. These elements contribute to an immunosuppressive environment that inhibits anti-tumor immune responses and promotes tumor progression. Tumor-associated macrophages (TAMs), microglia, and MDSCs facilitate tumor growth and immune evasion, while elevated Tregs further suppress anti-tumor immunity. GSCs release immunosuppressive factors such as CD95 and Programmed death-ligand 1 (PD-L1), which inhibit T-cell activity and immune surveillance. This combination of factors significantly undermines the effectiveness of T cell-based immunotherapies and accelerates tumor progression (73–75).

An immunosuppressive microenvironment in glioblastoma poses a significant challenge to current therapeutic approaches. While T cell-based immunotherapies have shown effectiveness in other cancer types, such as melanoma and lung cancer, they are less effective in treating glioblastoma due to the scarcity of tumor antigen-specific T cells and the highly immunosuppressive nature of the TME (76). Such unfavorable circumstances warrant the use of immune-stimulatory approaches, such as therapeutic cancer vaccines.

To overcome these barriers, multiple combination strategies have been explored.

Overcoming tumor-induced immunosuppression remains a critical challenge for effective cancer vaccination. Recent research has highlighted the potential of combination therapies, such as pairing vaccines with immune checkpoint inhibitors (e.g., anti-PD-1/PD-L1 and anti-CTLA-4 antibodies), to reverse T cell exhaustion and restore antitumor immunity (77, 78). Additionally, targeting immunosuppressive elements of the tumor microenvironment—such as regulatory T cells, myeloid-derived suppressor cells, and immunosuppressive cytokines (e.g., TGF-β, IL-10)—using small molecule inhibitors, neutralizing antibodies, or gene editing techniques has shown synergistic effects with cancer vaccines in preclinical studies (79, 80). Rational vaccine design now increasingly incorporates adjuvants or cytokines (e.g., GM-CSF, IL-12) to potentiate local immune activation further and overcome inhibitory signals within the TME. These integrated approaches are actively being evaluated in ongoing clinical trials and offer a roadmap for the next generation of cancer vaccine strategies.

It is hypothesized that the composition of TME-associated cells varies as the tumor progresses and therapeutic agents are used. In mouse models, selective BBB permeability has been shown to limit drug transport into the brain, complicating therapeutic efforts. However, glioma uses this immune privilege to evade immune surveillance, creating an immunosuppressive tumor microenvironment.

TME-modulating agents are expected to be more effective in reducing recurrence and improving survival outcomes. Single-cell profiling of tumor-associated cells and *in situ* scrutiny of tumor sub-regions revealed astonishing intra- and inter-tumor variability of immune cells, encompassing variations in exhaustion and activity status (81–84).

### 3.3 Need for effective treatments for brain cancer

Many of the standard-of-care treatments (radiation and chemotherapy) for primary brain cancers offer limited efficacy or have high levels of adverse side effects. Additionally, chemotherapies may have poor penetrance in the highly protected brain environment due to the BBB, and this inefficiency may contribute to the development of drug resistance (85, 86). Importantly, radio-chemotherapies have a strong detrimental effect on the overall well-being of immune cells despite enhancing immunogenicity (87–89). The use of steroids in glioblastoma patient management is warranted due to life-threatening edema. However, steroids limit the clinical benefit of immune checkpoint blockade in glioblastoma (90, 91).

Malignant diffuse primary brain cancers are aggressive in growth, infiltrate into and surround the normal brain tissue, and have a high propensity to reform, recur, and repopulate (92). The physical placement limits and confines commonly utilized in surgical resection techniques, combined with the unknown spread and infiltration of malignant cells throughout the brain environment, generally prevent the benefit of obtaining disease-free margins. Radiation therapy procedures, designed to be patterned and centered around the surgical cavity, are performed to avoid the proliferation of remaining rogue cells. The high sensitivity to radiation that the most common primary brain cancers demonstrate has motivated and oriented the traditional plan of over-irradiating involved brain fields extending from the resected sites (93). This extensive irradiation is performed at the cost of damaging and limiting healthy brain tissue performance. Currently, the selective approach of biopsying or surgical removal after therapy enhancement and then subjecting the remainder of the brain to additional radiation typically becomes the strategy of choice when the malady recurs, redevelops, or proliferates (94).

Over the last decade, several Phase 3 clinical trials have been developed and evaluated for using gene-modified or synthetic antigens to stimulate the growth, activation, and infiltration of a patient’s immune cells into the brain cancer microenvironment. These Phase 3 vaccine studies have represented the novel strategy of immunotherapy and share the true potential for being another layer within the standard of care radiotherapy and therapy to enhance treatment effectiveness significantly, aid in avoiding the accumulation of normal tissue toxicities, and possibly help to ameliorate, counteract, and reverse inherent and acquired drug resistance (95).

## 4 Clinical trials with a brain tumor treatment vaccine

Currently, 3,177 ongoing studies containing the keyword’ cancer vaccine’ are registered on clinicaltrials.gov, of which only 118 are in Phase 4. There are only 126 ongoing clinical trials for glioblastoma; none are in Phase 4, with the majority in Phases 1 and 2, and only 8 in Phase 3 (as of 02/26/2025). Instead of listing all trials with only superficial discussion, several pivotal trials poised to impact glioblastoma treatment were selected. All Phase 3 studies and the most recent clinical trials, are compiled in **Supplementary Tables 1** and **2**.

### 4.1 Current phase 3 clinical trials with a brain cancer treatment vaccine

Ongoing vaccine clinical trials investigate various formulations and delivery methods to identify effective immunotherapy strategies for glioblastoma patients. The complete list of results is available in **Supplementary Table 1**.

Among them, the DCVax-L (NCT00045968) is an investigational personalized DC vaccine that utilizes the patient’s own DCs to generate a vaccine that stimulates the body’s immune system to recognize and fight cancer cells. The pipeline includes collecting the patient’s DCs, exposing them to a tumor lysate derived from their tumor tissue, and then reinfusing these “educated” cells back into the patient to stimulate a tumor-specific immune response. More specifically, patient-derived monocytes are differentiated into DCs that are then matured, activated, and loaded with tumor-associated antigens obtained intraoperatively using a standardized collection kit. Such purified, antigen-loaded DCs, considered “educated”, are then administered via intradermal injection in the upper arm. Upon administration, the DCs convey tumor antigen information to immune effector cells, including T and B lymphocytes, thereby initiating a systemic antitumor response.

This trial was conducted in newly diagnosed glioblastoma patients, following post-resection surgery, and in combination with standard-of-care therapies (radiotherapy and temozolomide). Patients with recurrence were also included via crossover. The trial enrolled 331 participants to compare survival rates between those receiving DCVax-L alongside standard treatments versus control groups receiving standard care alone. The experimental group had a median overall survival of 19.3 months compared to 16.5 months for the control group, indicating the vaccine’s efficacy. Survival at 48 months was higher in the DCVax-L group than in the control group, and the trend continued at 60 months. In patients with recurrent glioblastoma treated with DCVax-L, the median overall survival was 13.2 months from relapse, compared to 7.8 months for the control group. Survival rates at 24 and 30 months post-recurrence were also higher in the DCVax-L group.

Additionally, patients with glioblastoma and methylated MGMT receiving DCVax-L showed improved survival compared to control patients (1). However, this study warrants some reflection on methodological issues related to the change in the primary endpoint, the extended accrual period, and the suboptimal validity of the external control population used as the comparison arm (2, 3).

The Rindopepimut (CDX-110) trial (NCT01480479) is investigating an experimental cancer vaccine designed to induce anti-cancer effects in patients with tumors expressing the EGFRvIII (96). The vaccine consists of a 14-amino-acid peptide spanning the EGFRvIII mutation, admixed with 150 μg of GM-CSF, and is administered intradermally into the thigh in 2–8 small-volume injections (totaling 0.8 mL) to minimize local adverse effects. The regimen consists of two priming doses followed by monthly boosters, which are aligned with temozolomide cycles (4). The vaccine stimulates the patient’s immune system to recognize and attack cells expressing mutated EGFRvIII receptors. This Phase 3 study involved 745 participants and compared the vaccine’s effectiveness with that of standard chemotherapy in control groups of newly diagnosed patients with EGFRvIII-positive glioblastoma.

The primary analysis of the ACT 4 study did not show a survival benefit for patients with EGFRvIII-positive glioblastoma and minimal residual disease (MRD) who received rindopepimut post-resection in combination with standard-of-care temozolomide chemotherapy compared to those who received a control. The median overall survival from randomization for patients with MRD who received rindopepimut in ACT IV was 20.1 months, consistent with the 20–22 month range reported in previous uncontrolled trials in the same population (4).

Humoral immune responses were monitored by ELISA using EGFRvIII peptide-coated plates, and patients were stratified *post hoc* into slow, moderate, or rapid antibody responders. Additionally, HLA typing and MGMT promoter methylation were assessed centrally for exploratory correlation analyses (4).

These clinical trials showed that Rindopepimut did not significantly increase survival in patients with newly diagnosed glioblastoma. However, the long-term survivor cohort upon rindopepimut treatment was notably larger than upon standard-of-care alone. Thus, a combination with other approaches might be required to show the efficacy of this immunotherapy in glioblastoma (5, 6).

One ongoing clinical trial, in phases 2 and 3, is the Proteome-based Personalized Immunotherapy for Malignant Brain Tumors (NCT01759810). The study, starting in 2012, involved 60 patients with inoperable recurrent glioblastoma multiforme who had failed two lines of standard chemotherapy and radiation therapy. Treatment was performed on bulk tumor (biopsy material), and the vaccine was administered alone, without concurrent standard-of-care treatment.

Participants were treated with allogeneic haploidentical hematopoietic stem cells (HSC), a DCs vaccine, and cytotoxic lymphocytes. HSCs were harvested from closely related donors after granulocyte-colony-stimulating factor (G-CSF) administration. Tumor and tumor stem cells were isolated from glioblastoma samples, and DCs were cultured from peripheral blood mononuclear cells (PBMC). A tumor sample thus provided tumor-specific antigens to prepare such a DC vaccine. Cytotoxic lymphocytes were obtained from peripheral blood after vaccine administration. There has been no conclusive evidence or updates since 2020, so the trial’s progress and ongoing status are uncertain.

The ADCTA vaccine (NCT04277221) is an autologous DCs-based vaccine (ADCTA-SSI-G1) designed for adjuvant immunotherapy in the standard treatment of recurrent glioblastoma. This vaccine was administered post-resection and in combination with standard-of-care therapy (Bevacizumab).

This vaccine utilizes DCs exposed to tumor antigens in a co-culture system to enhance the immune system’s ability to target and attack cancer cells growing in the brain. In the ongoing Phase 3 trial, the vaccine is administered subcutaneously near axillary or inguinal lymph nodes. The whole course consists of 10 doses: an initial double dose (2–4 × 10^7^ cells), followed by three bi-weekly and six monthly injections (1–2 × 10^7^ cells per dose). ADCTA is currently in Phase 3 to confirm the results of early-stage trials (7) and to evaluate the efficacy and safety of ADCTA immunotherapy combined with standard therapy versus standard therapy alone in patients with recurrent glioblastoma. Although the results from this ongoing trial are not yet publicly available, previous clinical studies have provided both immunological and clinical evidence supporting the potential benefit of this approach.

Specifically, efficacy data were reported in a Phase I/II clinical trial (Taiwan DOH/MA 0910072504, conducted at Chang Gung Memorial Hospital from 2003 to 2005) and a subsequent Phase II study (NCT02772094, 2005–2016), where overall survival (OS) was the primary endpoint. In the Phase I/II trial (n = 16), the median OS was 525 days (~17.5 months) with a 5-year survival rate of 18.8%, compared to 380 days and 0% in a matched historical control group (n = 63). Subgroup analysis revealed particularly favorable outcomes in patients with recurrent glioblastoma, who demonstrated a median OS of 966 days (~32 months) and a 5-year survival rate of 25%. Tumor regression on MRI and an increase in CD8^+^ tumor-infiltrating lymphocytes were also observed in some individuals following vaccination.

When both the Phase I/II and Phase II cohorts were combined (n = 59), the median OS was 22.9 months overall, with 21.8 months for newly diagnosed (n = 44) and 28.1 months for recurrent patients (n = 15). Although not statistically significant, this trend suggests that early administration of the vaccine, particularly in recurrent settings before radiotherapy, enhances therapeutic outcomes.

These clinical findings, together with the observed induction of antigen-specific CD4^+^/CD8^+^ T-cell responses and interferon gamma (IFN-γ) secretion (8), underscore the dual immunological and clinical activity of the dendritic cell-based vaccine in patients with malignant glioblastoma (97, 98).

Following previous studies demonstrating promising results with DC-based immunotherapies for glioblastoma, a Phase 3 clinical trial is underway to evaluate the safety and efficacy of an experimental hybrid DC vaccine (NCT06749925) in adult patients with newly diagnosed glioblastoma. This trial is conducted post-resection and in combination with standard-of-care therapy.

In a Phase 1/2 clinical trial involving 37 patients with glioblastoma or grade 4 astrocytomas, patients who received the hybrid DC vaccine showed significantly improved overall survival compared to historical controls. Vaccinated glioblastoma patients had an overall survival of 27.6 ± 2.4 months. Similarly, vaccinated grade 4 astrocytoma patients had an overall survival of 59.5 ± 15.9 months, longer than the 19.8 ± 2.5 months for the control group. Seven vaccinated patients are still alive, with survival ranging from 25.4 to 78.6 months since diagnosis (9).

Another clinical trial, NCT03149003, examines an investigational peptide cancer vaccine (DSP-7888) composed of peptides derived from the Wilm’s Tumor (WT1) protein, exhibiting immunomodulating and antineoplastic activities in combination with Bevacizumab, a monoclonal antibody targeting vascular endothelial growth factor, is approved by the Food and Drug Administration (FDA) (99, 100). This trial involves recurrent or progressive glioblastoma patients, treated post-resection, and the vaccine is administered in combination with chemotherapy (bevacizumab). With an enrollment of 221 patients, this study aims to establish the efficacy of the investigational vaccine, focusing on progression-free survival rates and overall survival outcomes. DSP-7888 (adegramotide/nelatimotide) is delivered intradermally: weekly for the first five doses, biweekly for doses 6–15, and every 4 weeks thereafter. Bevacizumab is administered intravenously every two weeks at 10 mg/kg. Mechanistically, DSP-7888 can induce WT1-specific cytotoxic T-lymphocytes against WT1-expressing tumor cells in HLA-A02:01+, HLA-A02:06+, and HLA-A*24:02+ individuals while stimulating a helper T-lymphocyte-mediated immune response against WT1-expressing tumor cells. DSP-7888 was well tolerated, with no dose-limiting toxicities, in patients with recurrent or advanced glioblastoma, and higher induction of WT1-specific cytotoxic lymphocytes was noted (10).

AV-GBM-1 is a therapeutic, patient-specific DC vaccine currently in Phase 3 development by AIVITA Biomedical (NCT05100641). This trial targets newly diagnosed glioblastoma patients following post-resection, in combination with standard-of-care therapies. The primary goal is to determine the therapeutic effect of AV-GBM-1 and its impact on the overall survival of patients with primary glioblastoma who have recently received standard therapy. DCs are collected from a patient’s blood, exposed to specific tumor antigens, and then reintroduced into the patient to stimulate the immune system to recognize and attack the cancer cells. For Phases 1 and 2, patients were recruited with resected primary glioblastoma and planned for concurrent chemotherapy and radiation. Resected tumors were used to establish a tumor cell culture, while whole blood was collected for PBMC isolation. In the current Phase 3 trial, the AV-GBM-1 vaccine is administered subcutaneously in combination with 500 µg GM-CSF, consisting of three weekly doses before adjuvant temozolomide, followed by monthly injections for up to 18 months (a total of up to 21 doses). Each dose contains ~2 million dendritic cells. If needed, cryopreserved cell lines can be re-expanded, or additional leukapheresis may be performed.

AV-GBM-1 vaccine was well-tolerated and increased the median progression-free survival. The study’s intent in Phase 3 clinical trials is to enroll approximately 726 patients for tumor retrieval, including 690 who are eligible for treatment at the time of randomization and have agreed to participate (11).

The TVI-Brain-1 is in a Phase 2b clinical study (NCT05685004) to treat newly diagnosed MGMT-unmethylated glioblastoma patients. Patients undergo surgical resection before vaccine preparation, and the immunotherapy is administered in combination with standard-of-care therapies, including radiotherapy and temozolomide.

This research study examines the potential application of T-cell-targeting neoantigens in patients with a specific subtype of glioblastoma. The TVI-Brain-1 belongs to vaccine-enhanced adoptive T-cell therapy, a personalized immunotherapy approach tailored to the individual patient’s tumor. The process of TVI-Brain-1 production involves: 1) Identifying antigens specific for cancer cells; 2) Collecting T cells from patients’ blood and triggering them with those antigens to become activated and trained to recognize the tumor antigens; 3) The vaccine with activated T cells is returned to the patient. Specifically, two subcutaneous vaccinations composed of attenuated autologous tumor cells are administered 7–14 days after surgery and spaced approximately one week apart.

Importantly, the vaccine is administered first to prime the immune system *in vivo*, typically within two weeks after tumor resection. Peripheral blood T cells are collected afterwards by leukapheresis and subsequently stimulated ex vivo with tumor-specific neoantigens, some of which were introduced during vaccination. These activated T cells are then reinfused following completion of standard chemoradiotherapy. A short course of low-dose interleukin-2 (IL-2) is administered after T-cell infusion to support T-cell function.

### 4.2 Newly developed early-phase clinical trials with a glioblastoma treatment vaccine

As several promising vaccine candidates progress from the laboratory bench to clinical trials, preventing glioblastoma recurrences via vaccination becomes more feasible. In addition, early-phase clinical trials add more options to the evolving toolkit for glioblastoma immunotherapy, emphasizing the transformative potential of these strategies. The complete list of results is available in **Supplementary Table 2**.

The isocitrate dehydrogenase 1 (IDH1) Vaccine Trial (NOA16), NCT02454634 (101) represents a pioneering effort to target a specific glioma mutation. This Phase 1 trial evaluated the safety and immunogenicity of an IDH1-specific peptide vaccine in 33 patients with newly diagnosed WHO grade 3 and 4 mutant IDH1-positive astrocytomas. The vaccine achieved its primary safety endpoint, with only minor vaccine-related adverse events reported. Furthermore, immune responses were observed in 93.3% of patients, which correlated with promising progression-free and overall survival rates. The vaccine, designed to target the mutant IDH1 enzyme, successfully induced cytotoxic T-cell responses and managed pseudoprogression, a phenomenon where treatment-related increases in tumor size reflect beneficial immune activation. These findings suggest that the IDH1 vaccine holds significant potential in inducing a targeted immune response, offering hope for preventing glioblastoma recurrence in patients harboring the IDH1 mutation (101). The vaccine consisted of a 20-mer peptide encompassing the IDH1 R132H mutation, emulsified in Montanide, and administered subcutaneously in combination with topical imiquimod. Patients received up to 8 doses at 2- to 4-week intervals, either as monotherapy after radiotherapy or in combination with temozolomide, depending on the treatment group. Although the NOA16 trial did not directly assess metabolic outcomes readouts such as D-2-hydroxyglutarate (D-2HG) levels or metabolic imaging, the observed immune activation, particularly IDH1-specific CD4^+^ T cell infiltration and pseudoprogression, suggests that immune pressure may indirectly affect the metabolic activity of IDH1-mutant tumor cells. Moreover, R-2-hydroxyglutarateR-2HG, the oncometabolite produced by mutant IDH1, is known to suppress antitumor immunity by impairing T cell infiltration and function, supporting the rationale for future studies investigating immunometabolic crosstalk and combination therapies with IDH inhibitors (102).

Advancements in mRNA and lipid nanoparticle technology, as seen with COVID-19 vaccines, are revolutionizing cancer treatment. This approach utilizes mRNA to educate the immune system about cancer and engages it in combating treatment-resistant cancers. Personalized mRNA vaccines, made from a patient’s tumor cells and delivered via lipid nanoparticles, address tumor heterogeneity and immune evasion by mimicking a viral threat to induce a targeted immune response (23).

A promising approach involves the use of RNA lipid particle aggregates (LPAs) in a Phase 1 clinical trial (NCT06389591) targeting adult glioblastoma patients with an unmethylated MGMT promoter. The RNA-LPA platform stimulates the immune system to reprogram the TME. By activating immune pathways such as RIG-I in stromal cells, RNA-LPAs promote the production of cytokines and chemokines that attract immune cells to the tumor site, ultimately enhancing the tumor’s susceptibility to immune attack. In this first-in-human Phase 1 study, patients are randomized to receive three doses of pp65 mRNA-loaded lipid particles (DP1) either before or after tumor resection, followed by monthly intravenous administrations of personalized RNA-LP vaccines (DP2) containing both pp65 and patient-specific tumor RNA, for up to 15 doses. This method is notable for stimulating systemic and local immune responses, creating a more conducive environment for immune cell infiltration into the tumor (95). Such an approach was successfully tested in preclinical studies using dog models of glioblastoma, which have demonstrated that mRNA vaccines offer significant survival benefits and establish new standards for treating aggressive brain tumors. These studies observed a rapid immune response and a shift towards an immune-responsive TME. The strategy involves creating a personalized vaccine from the patient’s tumor cells and utilizing engineered lipid nanoparticles that serve as both vaccines and immunomodulators (46).

In a recent research study, scientists focused on creating personalized mRNA vaccines to target neoantigens and tumor-associated antigens found in malignant brain tumors, using genomics-identified specific neoantigens and overexpressed tumor-associated antigens as their targets. The approach involved custom-designed mRNA vaccines that were intended to trigger an immune response specifically targeting these antigens. Their methodology involved a cancer immunogenomics pipeline, the Open Reading Frame Antigen Network (O.R.A.N.), to pinpoint and develop tailored mRNA vaccines. These vaccines were then administered in conjunction with immune checkpoint inhibitors or adoptive cell therapy. The approach appears promising, generating a potent anti-tumor immune response and resulting in an increase in tumor-infiltrating lymphocytes (TILs). Furthermore, the TME shifted significantly from “cold” to “hot,” indicating a positive impact on the body’s immune response to the tumor (47, 49).

OV therapy is an emerging cancer treatment that utilizes modified viruses to infect and destroy cancer cells while sparing normal tissue selectively. Such therapy is based on selective replication viruses, which can infiltrate, replicate, lyse human cancer cells, and spread within a tumor without causing damage to normal tissue. The OV approach combines direct tumor cell-specific cytotoxicity with the stimulation of an anti-tumor immune response (103). OV represents a unique class of therapeutics with distinct mechanisms of action, resulting from the biology of viruses of different origins and host-virus interactions. OVs naturally replicating in cancer cells exist, yet those genetically modified are used for therapeutic purposes to deliver specific treatments. Several OVs can infect and lyse cancer cells by inducing widespread cell death, presenting an opportunity to enhance the efficacy of traditional treatment modalities. Examples include the *Herpes simplex* virus (HSV), such as T-VEC, which has shown promise in melanoma treatment. T-VEC is administered directly into tumors or lymph nodes, exploiting the weakened antiviral defenses of cancer cells to cause cell death and stimulate an immune response against the released tumor antigens (104).

Unlike “hot” tumors, glioblastomas often have a “cold,” immune-suppressive microenvironment, which limits the effectiveness of conventional immunotherapies. OV therapy thus offers a promising solution by targeting these tumors with viruses that can replicate within and destroy cancer cells while transforming the TME into one more receptive to immune attack (105, 106). OV therapy is also particularly suitable for brain tumor treatment as it requires limited toxicity, and viruses targeting cancer do not harm other cells (107–109). The development of OV therapy for glioblastoma is progressing (110), with 22 OV therapy trials currently ongoing (as of February 26, 2025).

The results of a first-in-human phase 1 trial in 41 recurrent glioblastoma patients injected with an OV virus (CAN-3110) demonstrated that it retains the viral neurovirulence ICP34.5 gene transcribed under the transcriptional control of the nestin promoter to drive the expression selectively in nestin-overexpressing glioma cells but not in healthy differentiated tissue, thus conferring CAN-3110 with preferential tumor replication. In this Phase 1 trial, patients with radiologically suspected recurrent malignant glioma received a single intratumoral dose of CAN-3110 (rQNestin34.5v.2) during stereotactic biopsy, with study arms evaluating either monotherapy, pre-treatment with cyclophosphamide, or multiple injections over 120 days. CAN-3110 also has reduced neurotoxicity due to the deletion of the UL39 gene (encoding ICP6) and both endogenous copies of the γ_1_34.5 (ICP34.5) genes. These mutations thus enable rQNestin 34.5 to replicate specifically within tumor cells (111). No dose-limiting toxicities were encountered, and positive HSV-1 serology was significantly associated with improved survival, altered tumor PBMC T cell counts, peripheral expansion or contraction of specific T cell clonotypes, and tumor transcriptomic signatures of immune activation. These results provide human validation that intralesional Oncolytic herpes simplex viruses *(*oHSV) treatment enhances anticancer immune responses even in immunosuppressive tumor microenvironments, thus providing a biological rationale for the use of this oncolytic modality in cancers that are otherwise unresponsive to immunotherapy (NCT03152318) (44).

Another candidate, G47Δ, is a third-generation HSV-1 with a triple mutation that enhances replication and cytotoxicity in tumor cells while maintaining safety (112). In clinical use (UMIN000015995), G47Δ was administered intratumorally under MRI-guided stereotactic control at intervals of 5–14 days for the first doses. Then, every ~4 weeks, with each injection delivering 1 × 10^9^ pfu into up to three tumor coordinates using a dedicated low-dead-space injection needle (113). Preclinical studies of G47Δ have shown promise, with a one-year survival rate of 92.3% and a median overall survival of 20.2 months (114). The study noted an increase in CD4+ and CD8+ T-cell infiltration and a reduction in the number of FOXP3+regulatory T-cells in tumors after multiple doses of G47Δ, suggesting the potential of the therapy to transform the TME into a more immunologically recognizable one (115). However, the lack of persistent G47Δ in tumors and the challenge of pseudo-progression underscore the need for advanced technologies to analyze changes in the TME and develop additional methods to assess clinical benefit (115). Moreover, the biological heterogeneity of patients, including the diversity of IDH gene mutations, variability in MGMT promoter methylation, and potential selection biases related to participants’ good health, complicate the interpretation of results (116). A novel approach involving multiple injections and biopsies revealed increased T-cell infiltration, suggesting a transformation of the TME to a more immune-active one. However, the lack of long-term presence of G47Δ in tumors and the difficulty in distinguishing innate from adaptive T-cell responses to treatment efficacy remain unclear (116).

Combining oncolytic virotherapy with immune checkpoint inhibitors is another emerging strategy for treating glioblastoma. The CAPTIVE/KEYNOTE-192 study, documented under NCT02798406, conducted a Phase 1/2 trial exploring the combination of DNX-2401 and pembrolizumab, an anti-Programmed Cell Death Protein 1 (PD-1) antibody, for recurrent glioblastoma. The study aimed to assess the safety and effectiveness of combining these two treatments through a dose-escalation and expansion protocol. DNX-2401 was administered as a single intratumoral injection at escalating doses (5 × 10^8^ to 5 × 10^10^ viral particles), followed by pembrolizumab infusions every 3 weeks for up to 2 years or until disease progression occurred. When DNX-2401 infects and replicates within tumor cells, leading to tumor cell lysis, it releases tumor antigens and viral particles, stimulating an immune response. When combined with pembrolizumab, the therapy aims to further enhance the immune response by inhibiting immune checkpoints that cancer cells exploit to suppress immune activity. This dual approach promotes a more robust and sustained anti-tumor immune response. The multicenter trial of DNX-2401/pembrolizumab combination for recurrent glioblastoma involved the intratumoral injection of the vaccine, followed by systemic administration of the antibodies. The results indicated that the treatment was well-tolerated without dose-limiting toxicities. Although the primary efficacy endpoint was not achieved, the secondary endpoint of 12-month survival exceeded expectations, suggesting the potential for effective treatment of recurrent glioblastoma through combinational approaches. DNX-2401 targets cancer cells by selectively replicating in cells with defective retinoblastoma (Rb) pathway signaling, a common defect in many cancers, including glioblastoma. The results showed that the treatment met primary safety endpoints without dose-limiting toxicities and achieved a 12-month overall survival rate of 52.7%, surpassing the prespecified control rate, demonstrating significant clinical benefits (117, 118).

### 4.3 Summary of clinical trials approach for glioblastoma

Glioblastoma has a low mutational load, characterized by a limited number of non-synonymous mutations that can elicit a T-cell response. This lack of antigenic epitopes limits the immunogenicity of glioblastoma, making vaccines targeting tumor-specific epitopes crucial for triggering an immune response (119). Thus, it is a highly challenging malignancy characterized by its infiltrative nature and immune-resistant microenvironment.

As conventional therapies often fail to produce durable responses, novel treatment modalities are necessary. Recent advancements in cancer immunotherapy have sparked interest in harnessing the immune system to combat glioblastoma. Among these approaches, personalized vaccines have emerged as a promising strategy to elicit targeted immune responses against tumor-specific antigens. Personalized cancer vaccines are tailored to each patient’s genetic profile. By identifying patient-specific tumor antigens (neoantigens) through genomic sequencing and bioinformatics, vaccines can be designed to target these unique antigens, increasing specificity and potency. Research on the identification and deciphering of neoantigens, as well as the composition of cancer-specific immunopeptidomes, is currently advancing rapidly (47, 81, 120–124). This approach leverages the unique mutational landscape of individual tumors to prime the immune system to recognize and eradicate cancer cells. One promising idea is to generate tumor-reactive T cells in newly diagnosed glioblastoma patients, thereby transforming them into ‘hot’ tumors susceptible to immunotherapy approaches (125, 126).

Vaccine approaches in glioblastoma clinical trials aiming to harness the body’s immune system to target and destroy tumor cells with precision employ diverse strategies, from targeting specific tumor mutations, such as the IDH1 mutation in the IDH1 Vaccine Trial, to reprogramming the TME to enhance immune activity, as seen in RNA LPE and mRNA-based trials. The potential benefits of these vaccines are underscored by their ability to induce specific immune responses, which is evidenced by improved progression-free and overall survival rates in some trials, such as the DCVax-L. However, DCVax-L has sparked controversy in neuro-oncology due to its study design and concerns about using external and historical controls, the definition of trial endpoints, data transparency, and its regulatory approval status despite long-term studies (2, 61, 62).

The vaccine approaches in glioblastoma undoubtedly face significant challenges. The brain’s immunosuppressive microenvironment, glioblastoma tumors’ inherent heterogeneity, and the BBB substantially hinder effective vaccine delivery and function. Moreover, the early-phase nature of many of these trials means that while initial safety and immunogenicity profiles are promising, the long-term clinical benefits and impacts on survival are yet to be fully realized and understood.

Future trends in glioblastoma vaccine research will likely focus on combining vaccine therapies with other treatment modalities, such as chemotherapy, radiation, or newer immunotherapies like checkpoint inhibitors, to enhance efficacy. However, some combinations may not be advantageous, as it has been shown that standard temozolomide (TMZ) causes lymphopenia and T cell exhaustion. In contrast, radiation, which induces neo-antigen prevalence and stimulates immunogenic cell death, reduces both peripheral and local lymphocyte counts (17, 127). There is also a growing trend towards personalizing vaccine therapy—tailoring vaccines to the specific genetic profile of a patient’s tumor to improve outcomes. Continued research and clinical trials will be crucial to overcoming current limitations and establishing the role of vaccine therapies in the standard care of glioblastoma, leading to more robust, targeted, and effective treatment regimens for this aggressive cancer.

## 5 Challenges of utilizing cancer vaccines

The utilization of cancer vaccines faces significant challenges that can be broadly categorized into immunological challenges, vaccine composition, and regulatory hurdles imprinted into the brain TME. These obstacles complicate the path from research and development to clinical application and widespread use. An analysis of current clinical trials highlights the limitations of vaccine testing, particularly in validating patient responses in phase 3 studies. Additionally, reviewing newly developed early-phase clinical trials allows for a discussion of recent approaches to vaccine testing and their potential benefits for patients. This provides valuable insights for patients with glioblastoma regarding the research advancements needed to improve outcomes. A thorough understanding of the mechanisms, efficacy, and patient responses associated with the various brain tumor treatment vaccines mentioned in clinical trials is essential for proper evaluation.

Innovative approaches in early-phase trials, such as the IDH1 Vaccine Trial (101), RNA-LPAs (46), and mRNA-based vaccines (16, 47, 49, 128), can potentially target specific tumor mutations and reprogram the tumor microenvironment. These strategies aim to shift the immunological status of tumors from “cold” to “hot,” enhancing the overall immune response. While these are promising, their long-term effectiveness and impact on survival rates will need further validation in subsequent trials.

Thus, these clinical trials represent a dynamic and diverse exploration of immunotherapy strategies against glioblastoma, each with unique mechanisms and potential benefits. Ongoing research and forthcoming results will be crucial in determining the most effective approaches and integrating them into comprehensive treatment protocols for glioblastoma.

### 5.1 Challenges of utilizing cancer vaccines

**Figure 4:**
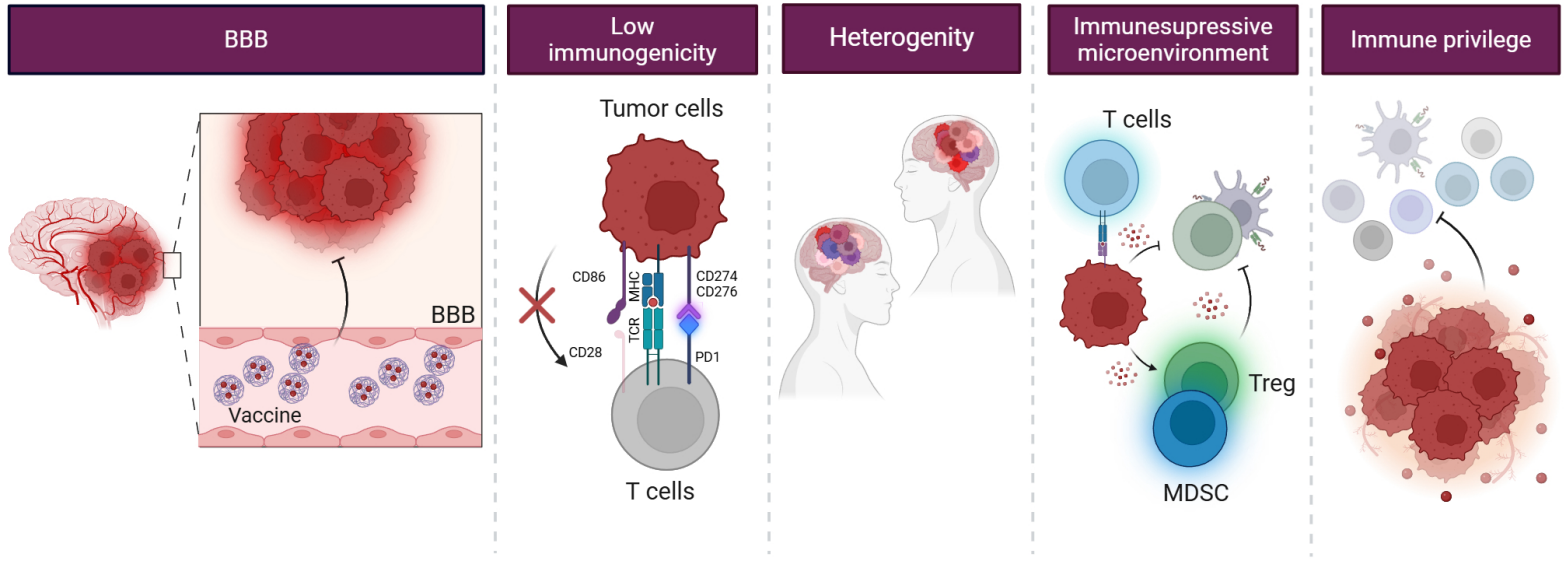
Challenges in glioblastoma treatment. Glioblastoma poses a challenge for immunotherapy due to its limited immunogenicity, primarily affected by the BBB and the immunosuppressive TME. The BBB restricts the entry of therapeutic agents into the brain, making it challenging to deliver immunotherapy to the tumor site. Additionally, the TME organizes an immunosuppressive environment, protecting GSCs from recognition and immune attack. They also often mimic their antigens, avoiding detection and removal by the immune system. Moreover, they recruit immunosuppressive cells, such as MDSCs and Treg cells, and secrete immunosuppressive cytokines, which inhibit the activation and function of immune cells.

Immunological challenges significantly hinder effective immune responses against tumors due to the complex interaction between the immune system and cancer cells and the unique characteristics of the TME. The intricacies of these interactions are particularly pronounced in the context of brain tumors, where physiological barriers present additional challenges to vaccine efficacy (**Figure 4**).

#### 5.1.1 Immunological and molecular complexity

Each cancer is molecularly and immunologically unique. Identifying antigens that provoke an immune response strong enough to prevent cancer cell resurgence while sparing normal tissues is thus critical yet highly challenging. However, the task is constrained by tumor heterogeneity, suboptimal immunogenicity instilled by the TME, and the tolerance of the immune system to self-antigens (129). Overcoming this tolerance and eliciting robust reactions against tumor-specific antigens are essential for vaccine efficacy. Cancer’s heterogeneity adds further complexity to vaccine development. Each tumor may exhibit a distinct array of antigens, making the creation of a universal vaccine a daunting task. Addressing this variability requires a personalized approach to vaccine design. High-throughput technologies can expedite the discovery of neoantigens, while Artificial Intelligence (AI) and machine learning facilitate data analysis and prediction, thereby aiding vaccine development (81, 121, 122).

The TME of glioblastoma is particularly challenging due to the presence of immunosuppressive cells, such as TAMs and MDSCs, which significantly reduce the functionality of APCs and T cells. TAMs, often polarized to an M2 phenotype in the tumor milieu (130), promote tumor growth and suppress anti-tumor immunity by releasing cytokines that inhibit the cytotoxic activities of T cells. MDSCs contribute to the immunosuppressive environment by producing arginase, nitric oxide, and reactive oxygen species, further inhibiting T cell function and proliferation. These factors collectively result in a TME that protects the tumor from immune surveillance and complicates the efficacy of cancer vaccines by limiting the immune system’s ability to mount a robust and effective response against the tumor antigens (41, 42).

#### 5.1.2 Physiological barriers and clinical translation

The CNS poses unique challenges due to its immune privilege—a unique immune system’s policing—and the presence of the BBB. These physiological barriers limit immune surveillance and hamper the efficacy of immunotherapies, including cancer vaccines. The BBB effectively protects the brain from pathogens and other foreign substances, but also obstructs the delivery of therapeutic agents. This dual role of the BBB makes glioblastoma therapy particularly challenging, especially since many chemotherapeutic drugs have low cerebrospinal fluid-to-plasma ratios (131). Despite the BBB disruption in some tumor areas, the intact BBB in infiltrative regions prevents the distribution of drugs to invasive tumor cells (132).

Most conventional BBB-opening strategies, such as convection-enhanced delivery (133, 134), intracranial implantation (135, 136), and deep-brain stimulation (137, 138), are challenging to apply in the clinical setting due to their broad, non-specific modulation of the BBB, which can damage normal brain tissue (139), and various non-invasive strategies are being explored to cross the BBB and deliver drugs effectively.

Strategies such as nanoparticle-based delivery systems or focused ultrasound are being investigated to enhance the delivery of vaccines and drugs to the brain. Passive transcytosis enhances drug delivery through paracellular or transcellular pathways by modulating BBB permeability. By modulating tight junctions, paracellular routes can be enhanced, improving drug delivery to brain tumors (140). The transcellular pathway, preferred for larger molecules, often employs lipid nanoparticles that can be modified to target and effectively deliver drugs across the BBB (141). To address these physiological barriers, refined delivery platforms have been developed. Ligand-conjugated nanoparticles targeting receptors such as transferrin, insulin, or folate enable receptor-mediated transcytosis across the BBB. Cell-membrane-coated systems, based on erythrocytes, tumor cells, or immune cells, provide immune evasion, prolonged circulation, and tumor-specific targeting. Additionally, stimuli-responsive nanoparticles activated by near-infrared light or ultrasound enable transient and localized opening of the BBB, thereby minimizing off-target toxicity (142).

Intranasal administration offers another promising route, bypassing the BBB via the olfactory and trigeminal nerves. This method efficiently delivers drugs to the brain while minimizing peripheral exposure. However, it faces challenges such as variability in nasal anatomy, mucociliary clearance, and health conditions like colds or allergies (143).

Ligand conjugation is an active targeting strategy that uses ligands with high specificity for receptors on brain endothelial cells, such as transferrin or insulin-modified nanoparticles (144). This approach facilitates targeted drug delivery across the BBB. Ligands enable transcytosis, allowing drugs like TMZ and liposomes to cross the BBB and reach glioblastoma cells (145).

Additionally, cell membranes are increasingly used in drug delivery systems for brain targeting due to their natural functionalities and ability to interact with various substrates. Membrane coatings, such as those found in red blood cells or tumor cell membranes, enhance circulation and tumor targeting (146). External stimuli such as near-infrared light (147), ultrasound (148), and electroacupuncture (149) can transiently disrupt the BBB, enabling targeted drug delivery with minimal damage. Other strategies, such as polysorbate-80 (PS-80) and engineered AAV9, also facilitate BBB crossing (150, 151).

These technologies are especially promising for mRNA and peptide-based glioma vaccines, which benefit from lipid nanoparticles, redox-sensitive carriers, and viral vectors engineered for CNS delivery. To address limitations in antigen delivery and immunogenicity, recent advances have focused on developing innovative delivery platforms for cancer vaccines. Nanoparticle-based systems, including liposomes, polymeric nanoparticles, and biomimetic extracellular vesicles (EVs), are being engineered to improve the stability, targeted delivery, and cellular uptake of tumor antigens (49, 152). These platforms can be functionalized with ligands or antibodies to enhance selective targeting of dendritic cells or other antigen-presenting cells *in vivo*, thereby maximizing antigen presentation and immune activation while minimizing off-target effects. Intratumoral or intracranial delivery approaches, including the use of Ommaya reservoirs or convection-enhanced delivery, have also shown promise in bypassing the blood-brain barrier and achieving high local concentrations of vaccine components, as recently demonstrated in preclinical glioma models (142). Such strategies not only enhance vaccine efficacy but also reduce systemic toxicity, representing a crucial direction for future research and clinical translation.

Co-delivery strategies incorporating immunostimulatory agents or adjuvants that modulate the tumor microenvironment may further enhance immune activation and counteract glioma-induced immunosuppression (142).

In conclusion, developing effective cancer vaccines for brain tumors involves activating a robust immune response and maintaining the delicate immunological balance within the CNS. Advances in technology and a better understanding of the brain TME are driving the development of innovative strategies that aim to overcome these challenges. While promising, these strategies introduce complexities and potential risks that must be carefully evaluated in clinical settings.

### 5.2 Immunological mechanisms

The shift towards neoantigen-based vaccines and other novel compositions in glioblastoma treatment reflects a deeper understanding of the immunological mechanisms that underpin effective cancer immunotherapy. These novel vaccine strategies enhance antigen presentation and T-cell activation in an environment traditionally characterized by immune evasion. These vaccines aim to overcome central tolerance and provoke a more potent immune response by targeting neoantigens (21, 49).

Moreover, the efficacy of these approaches is often augmented by the strategic inclusion of adjuvants that can modulate the TME to be more conducive to immune activity. For instance, the addition of toll-like receptor agonists to vaccine formulations has enhanced DC activity and T-cell priming, thereby improving the immunogenic landscape of glioblastoma (153).

Further, addressing the challenge to effective antigen presentation posed by the BBB, recent studies have explored nanoparticles and other delivery systems that can penetrate the BBB more efficiently. This strategy ensures that the vaccine components reach the tumor site and can exert their immunomodulatory effects directly within the CNS, thus potentially increasing the clinical viability of these innovative therapeutic approaches (154, 155).

In summary, the comparative analysis of vaccine compositions highlights progress from conventional to more tailored strategies. It highlights the crucial role of understanding and manipulating immunological mechanisms to improve the efficacy of glioblastoma vaccines.

## 6 The complexity of cancer vaccine components

The glioblastoma vaccine development landscape has evolved from traditional vaccine strategies, primarily focusing on broad-spectrum tumor-associated antigens (TAAs), to innovative approaches incorporating patient-specific neoantigens (20, 81, 121, 122, 125, 156). Conventional vaccine strategies have leveraged antigens such as EGFRvIII (4, 6), a mutation prevalent in a subset of glioblastoma patients, and have demonstrated modest clinical benefits. These approaches, while foundational, often suffer from limitations in inducing a robust and durable immune response due to the heterogeneous and immunosuppressive nature of the glioblastoma microenvironment.

In contrast, recent advancements in vaccine composition have led to the emergence of neoantigen-based vaccines, which promise enhanced specificity and immunogenicity. Neoantigens, unique to individual tumors and arising from tumor-specific mutations, present a therapeutic target that is less likely to induce tolerance and more capable of eliciting a strong cytotoxic T-cell response (47, 81, 121, 125). Phase 1 trials employing these novel vaccine strategies have begun to show potential in generating personalized immune reactions that could improve clinical outcomes in glioblastoma treatment.

**Figure 5:**
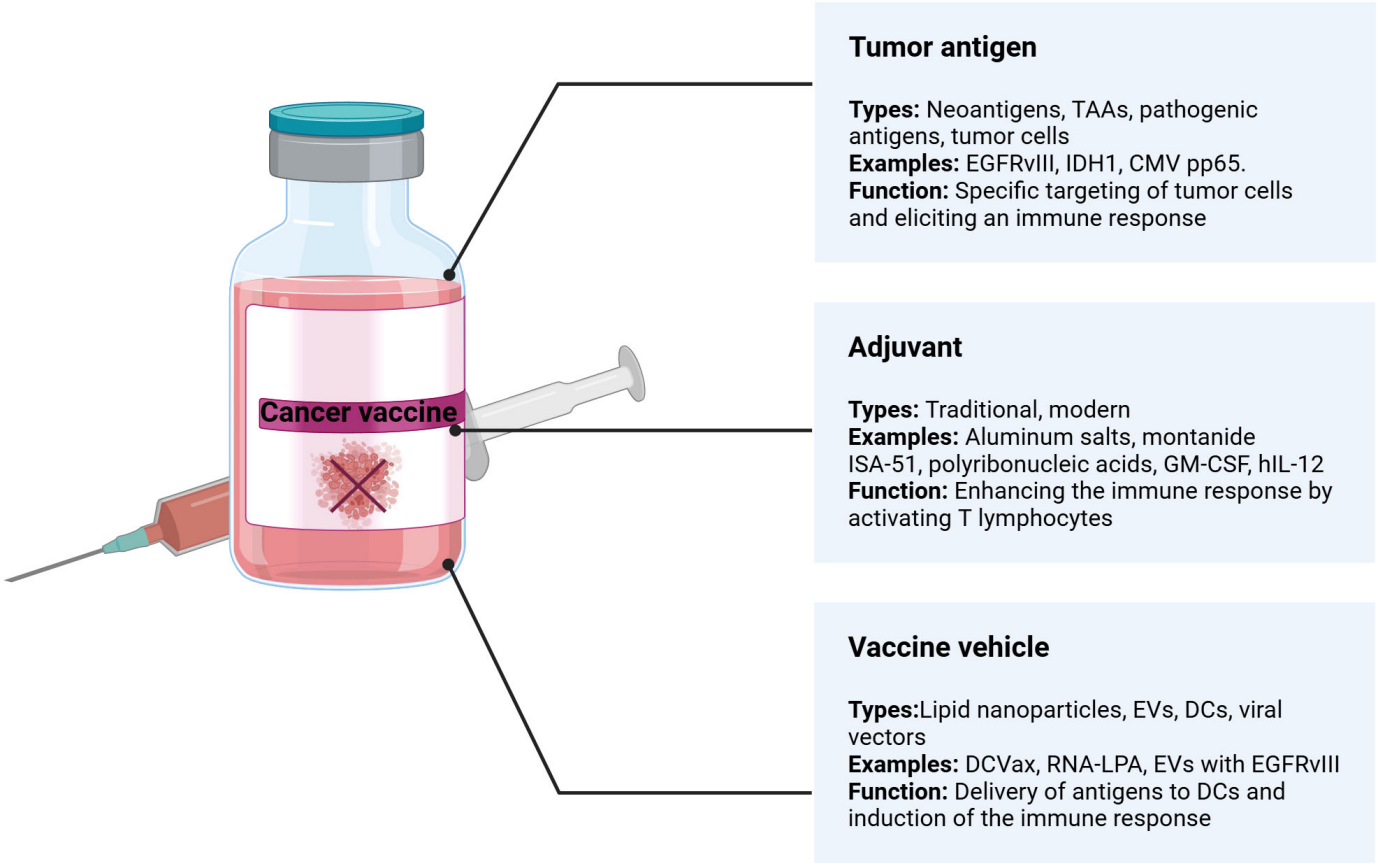
Key components of cancer vaccines.

Cancer vaccines comprise three main components: a tumor antigen, an immunological adjuvant, and a vehicle or carrier (**Figure 5**). The success of these vaccines hinges on selecting each element appropriately, making the optimization process complex.

### 6.1 Tumor antigen

Vaccines often contain well-defined tumor antigens delivered as DNA, RNA, proteins, or peptides. Well-defined antigens offer specificity, targeting specific molecular pathways and minimizing off-target effects and the need for synthetic production. Next, synthetic antigens can be consistently manufactured in large quantities, ensuring uniformity and availability of the vaccine. The ability to customize DNA and RNA vaccines to target specific mutations or adapt to new ones is a significant advantage. However, compared to the proteome-to-peptidome strategy, the use of a limited number of well-defined antigens in DNA or RNA vaccines raises concerns about their ability to elicit a strong immune response capable of addressing the heterogeneity of brain tumors (156). Antigens used in cancer vaccines are broadly classified into neoantigens and TAAs (157, 158).

Neoantigens (81, 121, 156) are novel proteins arising from tumor-specific mutations not present in normal tissues, making them ideal targets for personalized cancer vaccines due to their high immunogenicity and specificity (156). For example, the clinical trial of TVI-Brain-1 (NCT05685004) explores adaptive T-cell targeting of neoantigens in glioblastoma, harnessing the body’s immune response to target specific mutations unique to each tumor. Neoantigens, deriving from somatic mutations exclusive to tumor cells, present a particular target for immunotherapy. EGFRvIII is a predominant neoantigen identified in glioblastoma, originating from a deletion mutation. Initial promising results from early-phase clinical trials highlighted its potential; however, subsequent phase 3 trials (e.g., the multicenter study on rindopepimut (4, 6)) revealed limitations due to the loss of antigen expression post-treatment, an outcome possibly stemming from cancer immunoediting processes, underscoring the challenges of solely relying on neoantigens due to their variability and potential for disappearance in recurrent disease stages.

Similarly, a mutation in IDH1, found in specific glioblastoma subtypes, is being explored in clinical trials of peptide vaccines to prevent tumor progression, thereby eliciting mutation-specific T-cell responses (101).

TAAs are proteins typically overexpressed in tumor cells compared to normal cells. They include proteins like Melanoma-Associated Antigen 1 (MAGE-1), human epidermal growth factor receptor 2 (HER-2) (158, 159), and survivin, an inhibitor of apoptosis, targeting of which aims to elicit cytotoxic T-cell responses, which shows the potential of such antigens to elicit an immune response (45). However, their effectiveness in prolonging survival remains under investigation in combination with other therapeutic modalities to achieve significant clinical benefits.

The immunosuppressive microenvironment of brain tumors, particularly glioblastoma, can inhibit the effectiveness of immune responses initiated by the vaccine. So, delivering genetic material into cells efficiently, especially across the BBB, and navigating regulatory and developmental hurdles are significant challenges. Thus, using well-defined TAAs for cancer vaccines in treating brain tumors offers specificity, safety, and scalability advantages. However, addressing tumor heterogeneity, immune evasion, and delivery barriers is crucial for successful implementation.

Alternative Antigen Sources are Pathogen-derived antigens: The investigation into CMV-specific antigens in glioblastoma illustrates the exploration of such exogenous antigens. Although the presence of CMV in glioblastoma is debated, clinical trials targeting CMV antigens have proceeded, with some showing potential for inducing robust immune responses. Vaccines targeting CMV antigens, such as pp65, were proposed as virus antigen-loaded DC vaccines (55). The discovery and validation of new antigens through genomic and proteomic studies are crucial for continuously advancing tumor immunotherapies. High-throughput sequencing, sophisticated mass spectrometry techniques (MS), and advanced bioinformatics tools are crucial for identifying novel antigens derived from non-canonical sources, including non-coding regions, alternative splicing, and epigenetic modifications (121, 122, 156). These discoveries are rapidly translated into clinical trials, testing new vaccine formulations and delivery methods to enhance their therapeutic efficacy and patient-specific responsiveness.

Antigens from alternative splicing and transposable elements are increasingly recognized for their potential to provide novel and unique antigens. Aberrant splicing events and transposable elements can generate peptides not present in normal cells, providing new targets for vaccine strategies. However, epigenetic therapy (e.g., DNMT1 and HDAC inhibitors) can generate tumor-specific transposable element-derived antigens in glioblastoma but also activate transposable elements in normal cells, requiring careful prioritization of candidate antigens and perhaps using cancer cell-specific agents to create tumor-enriched antigens in glioblastoma cells ex vivo as OVs may offer a strategy that eliminates normal cell response (157, 160).

It is recognized that whole tumor cell lysates offer a promising alternative due to their ability to induce more robust and durable immune responses by presenting a broader array of antigens (161). This approach minimizes the risk of tumor immune escape by delivering a broader range of antigens, thereby overcoming the limitations observed with vaccines based on single antigens (162). The heterogeneity and adaptive nature of brain tumors make cancer cell-derived lysates particularly appealing, enhancing the likelihood of a robust immune response. The complexity of lysates, the risk of autoimmunity, the need for personalized manufacturing processes, and the difficulty in obtaining tumor material for vaccine production are the standard arguments against such a strategy (161). However, current technology enables the controlled processing of biological specimens, and brain tumor biopsy biomaterial proves to be a source of cancer cells that can be isolated, cultivated, and modified through immunoreactivity. Still, concerns persist about the duration and specificity of immune responses, as well as the tumor’s ability to evade them. Ongoing research and clinical trials aim to refine the use of tumor lysate vaccines, potentially by combining them with other immunotherapeutic strategies to enhance their effectiveness and address these limitations (163). For brain tumors, such strategies have reached clinical trial level 3 (1, 164). Alternatively, cellular components such as a mitochondrial fraction or EV-derived biomaterial can serve as a vaccine platform to effectively deliver antigens to DCs and induce potent antitumor immunity in preclinical models (24, 165, 166).

However, despite their immunogenic potential, both tumor lysates and EVs can also carry factors that contribute to tumor progression or immune suppression, which has raised concerns regarding their safety and therapeutic consistency.

Due to their ability to capture both tumor-specific and tumor-associated antigens, tumor lysates and EVs are widely utilized as sources of tumor antigens for cancer vaccine development, thereby addressing tumor heterogeneity and immune escape (167). Tumor lysates, generated from disrupted tumor cells, provide broad poly-antigenic stimulation and have been used in clinical trials for vaccines against glioblastoma and melanoma. However, both lysates and EVs can also harbor immunosuppressive cytokines (e.g., TGF-β, IL-10), growth factors, and regulatory RNAs that may promote tumor progression, modulate the tumor microenvironment (TME), or suppress anti-tumor immunity (168).

EVs, particularly exosomes, have emerged as promising alternatives due to their selective and stable antigen-carrying capacity, inherent biocompatibility, and efficient delivery to dendritic cells; examples include EV-based vaccines investigated for melanoma, colorectal, and lung cancers (169). Nonetheless, cancer-derived EVs can transport immunosuppressive molecules, such as PD-L1 (170), regulatory miRNAs (171), and tumor-promoting proteins (172).

To address these limitations, recent strategies have leveraged therapeutic stress (e.g., immune checkpoint blockade, oncolytic viruses, chemotherapy) to reprogram the antigenic content of lysates and EV cargo (173). Such stressors can enrich for immunogenic peptides, including those derived from noncoding RNAs and defective ribosomal products (DRiPs), while reducing tumor-promoting and immunosuppressive components (174–177). For example, EVs derived from oncolytic virus-treated tumor cells exhibit increased immunogenicity and reduced oncogenicity, as demonstrated in preclinical glioblastoma models (178–180).

Although alternative antigen sources, such as purified MHC-peptide complexes or synthetic peptides, offer greater specificity, their application is limited by technical complexity and the challenge of identifying personalized antigens, particularly in tumors with low mutational burdens. Therefore, despite these caveats, lysates and EVs—especially when manipulated by therapeutic stress—are emerging, versatile platforms for generating diverse, patient-specific cancer vaccines (168, 174).

The growth of tumors is controlled by the immune system’s recognition of MHC Class I and Class II peptides by CD8+ and CD4+ T cells. To date, immune checkpoint inhibitors have not shown success in clinical trials for glioblastoma. However, targeted immunotherapies using MHC Class I neoantigens or tumor-associated antigens have shown potential therapeutic benefits in specific patients, especially when the treatment matches a patient’s immunopeptide expression profile (122, 129). Recent advances in MS and bioinformatics have facilitated the exploration of the glioblastoma immunopeptidome, revealing potential targets for immunotherapy (13, 120). The immunopeptidome, which comprises peptides presented by MHC molecules, originates from various sources, including cells, tissues, and biofluids. EVs have attracted attention for their ability to modulate immune responses by carrying MHC molecules and associated antigens. In doing so, they serve as vehicles for vaccines (181). Preclinical evidence suggests that ex vivo–engineered EVs derived from glioblastoma patient-derived cells can carry key immunologically relevant components, such as PD-L1 and MHC molecules (170). While this approach holds promise for enhancing the efficacy of cancer vaccines in clinical trials, its effectiveness is currently limited by challenges in achieving precise and consistent formulation.Used in the Proteome-based Personalized Immunotherapy (NCT01759810) trial, this innovative approach targets a broad spectrum of antigens derived from tumor proteomes, potentially overcoming the limitations of antigen variability and tumor heterogeneity.

### 6.2 Adjuvant type

The development of cancer vaccines for brain tumors like glioblastoma encounters numerous immunological challenges, given the stringent requirements for clinical efficacy and the immunosuppressive TME typical of brain cancers. A key strategy to overcome these hurdles is the use of adjuvants in vaccine formulations, which enhance antigen immunogenicity, a crucial step in initiating robust immune responses against tumors. Traditional adjuvants such as aluminum salts enhance humoral immunity but are less effective in inducing the cellular immunity critical for combating solid tumors (13).

Modern adjuvants, including water-in-oil emulsions and nanoparticles, are designed to provoke a more vigorous T-cell response by including danger signals that activate the immune system. These adjuvants are particularly valuable in targeting the cells within the highly immunosuppressive environment of the CNS, steering the immune response toward a Th1-type cellular profile that fosters cytotoxic T cells capable of attacking tumor cells. Recent trials have proposed using Montanide ISA-]51 (45, 182), an oil-based adjuvant that forms a depot at the injection site, thereby prolonging antigen exposure through a slow-release system and enhancing immune activation over time. It was used in trials where the combination of SurVaxM in Montanide ISA-51 was found to be a safe and well-tolerated regimen (45).

Because adjuvants contribute to sustained antigen release and exposure, they ensure continuous immune stimulation, addressing the need for recurring tumors such as glioblastoma. Their flexible application allows for tailored immune responses that are synergistically combined with other therapeutic strategies, such as checkpoint inhibitors, thereby enhancing overall treatment efficacy.

OVs are a form of adjuvant, as OV-induced oncolysis alters the TME, promoting proinflammatory pathways and activating resident and newly recruited immune cells through exposure to viral and possibly tumor antigens. Data from a first-in-human phase 1 clinical trial with CAN-3110—an oHSV (NCT03152318) showed that the cohort with HSV1-positive serology was characterized by prolonged survival, associated with differences in T cell clonotype metrics and tumor transcriptomic signatures linked to immune activation programs. OV immunotherapy can be further combined in early clinical trials with checkpoint inhibitor adjuvant therapy, which has sound effects. The combination of intratumoral OV (DNX-2401) followed by anti-PD-1 antibody (pembrolizumab) was safe and associated with a notable survival benefit in select patients (NCT02798406) (118). While these results support the therapeutic potential of intralesional OV-based strategies, they also highlight the importance of managing local immune-related toxicity. Cerebral edema is a recognized adverse event following intratumoral administration of oncolytic viruses in glioma therapy. In the DNX-2401 clinical trial, brain edema occurred in 37% of patients, with 16% experiencing serious Grade 3 or higher events. These were effectively managed with corticosteroids or bevacizumab, and no cases required surgical intervention. Similarly, in preclinical studies supporting the NCT03152318 trial of rQNestin34.5v.2, tumor-associated brain edema was observed in several treated mice. Histopathological analyses revealed edema often accompanied by necrosis or hemorrhage, and in some cases, these changes were considered the likely cause of death. Although derived from murine models, these findings were critical in shaping clinical dose-escalation protocols and ensuring strict safety monitoring, particularly for tumors located near eloquent brain areas (118, 183).

These findings provide human immunological and biological evidence supporting intralesional oncolytic treatment modalities to change the immunosuppressive TME into one more favorable for immunotherapy, providing broad relevance for the therapy.

Another approach was tested by combining two checkpoint inhibitors - ipilimumab (anti-CTLA4) and nivolumab (anti-PD1) to reverse T-cell exhaustion and enhance vaccine efficacy in the immunosuppressive TME. Although these immune checkpoint inhibitors have not yet been tested in combination with the vaccine in clinical trials, they were found to be safe and well-tolerated (48, 50).

In the SurVaxM trial for glioblastoma, vaccination was experimentally combined with fixed doses of recombinant granulocyte macrophage-colony stimulating factor (GM-CSF), a commonly used immunomodulator (184), demonstrating safety and favorable T-cell immune responses.

A clinical trial using Human interleukin-12 (hIL-12), a cytokine with anticancer activity, demonstrated acceptable tolerability of regulated hIL-12, with encouraging preliminary results that support an immunological antitumor effect of hIL-12. It utilized viral vectors (Ad-RTS-hIL-12) to overcome the systemic application limitation caused by toxic inflammatory responses, revealing mostly pseudoprogression accompanied by increased TILs that produce IFN-γ and PD-1 (184). Interestingly, GM-CSF added to the T-vec vaccine against melanoma helps kill cancer cells and stimulates the immune system to target the tumor (185). Thus, research into novel adjuvants with improved safety profiles and enhanced immune-stimulating abilities is promising, potentially enhancing the efficacy of glioblastoma vaccines.

Cytokine production enhances DC activation, and as an adjuvant to peptide vaccines, polyriboinosinic-polyribocytidylic acid-poly-L-lysine carboxymethylcellulose (poly-ICLC) has been demonstrated to be safe and capable of eliciting immunological activity to boost therapeutic responses (126). The personalized antigen approach was based on mutations identified by analyses of the transcriptomes and immunopeptidomes of the individual tumors. The approach was feasible, and vaccines that included poly-ICLC and GM-CSF as adjuvants displayed favorable safety and strong immunogenicity, eliciting sustained responses of central memory CD8+ T cells or inducing predominantly CD4+ T cell responses of T helper 1 type against predicted neoepitopes (126, 186–188).

Delivering adjuvant-enhanced vaccines across the BBB poses another significant technical challenge. Ensuring that adjuvants can effectively reach the tumor site and stimulate local immune responses within the brain is crucial for their efficacy (139).

In response to these challenges, emerging therapeutic approaches such as neoadjuvant designs and anti-PD-L1 therapies offer promising avenues. After surgical resection, neoadjuvant therapy primes the immune system against tumor-specific antigens, potentially reducing tumor mass and altering the TME (49). This approach provides direct insights into the vaccine’s impact, informing subsequent treatment strategies. Anti-PD-L1 immunotherapy, noted for its efficacy in other cancers, enhances the visibility of tumor cells to the immune system, improving the immune response against the tumor. However, it must be adeptly managed to address BBB penetration and variable PD-L1 expression among tumors (154).

Integrating adjuvants into vaccine formulations presents several challenges. Overstimulation can lead to inflammation and autoimmunity, which are particularly concerning within the CNS. So, while promising, these strategies introduce complexities and potential risks that must be carefully evaluated in clinical settings to ensure tangible benefits for patients with brain tumors.

Combining cancer vaccines with other immunotherapy agents, such as immune checkpoint inhibitors or cytokine therapies, can enhance their effectiveness by countering immune suppression in the TME and boosting the immune response (17, 189).

### 6.3 Vaccine vehicle

The choice of delivery systems is particularly critical for glioblastoma vaccines. While prophylactic vaccines are often administered via less invasive routes, such as oral, transdermal applications, or intratumoral injection, to minimize side effects, therapeutic vaccines for glioblastoma require more direct delivery methods. These include subcutaneous, intradermal, intraperitoneal, and intranodal injections to ensure optimal antigen presentation by APCs. Innovative delivery techniques, such as ultrasound-guided lymph node injections, are being explored to enhance the efficacy and durability of antitumor responses specific to glioblastoma (13, 120).

The selection of an appropriate delivery system for brain tumor vaccines is a crucial factor that directly impacts the efficacy and safety of the treatment. Various delivery vehicles or systems, each with unique mechanisms, are used to ensure that the antigens and adjuvants in the vaccine can effectively reach and stimulate the immune system; this is particularly important in treating primary, recurrent, and metastatic brain tumors, where overcoming the brain’s protective barriers and localized immune suppression are significant challenges. When considering the advantages, specific delivery systems, such as DC-based vaccines used in the DCVax clinical trial (NCT00045968 (3, 190),) or viral vectors carrying adjuvants (184), can enhance the presentation of antigens to the immune system, ensuring that the antigens are recognized and processed effectively, thus being crucial for initiating a strong and targeted immune response against brain tumor cells.

Additionally, advanced delivery systems like nanoparticle-based or liposomal carriers can be engineered to cross the BBB, targeting the delivery of antigens directly to the tumor site or areas of metastasis. Combining RNA technology and LPA in the trial (NCT06389591 (49),) demonstrates the potential of personalized vaccination with an efficacious delivery profile. This targeted approach helps maximize the vaccine’s impact while minimizing systemic side effects (46, 95). Moreover, some delivery systems enable the controlled release of antigens and adjuvants, maintaining sustained immune stimulation over extended periods, which is particularly beneficial for treating slow-growing tumors or managing recurrent brain tumors by actively engaging the immune system.

Furthermore, modern delivery vehicles can be designed to carry multiple types of antigens and adjuvants, facilitating combinatorial therapies that target different aspects of tumor biology, leading to a more comprehensive immune attack on the tumor. On the other hand, there are also disadvantages to consider. Developing and manufacturing specialized delivery systems, especially those designed to cross the BBB or target specific areas within the brain, can be complex and costly (191), limiting the accessibility of such treatments and increasing the time required to bring them to clinical use. Additionally, while targeting the brain, there is a risk that the delivery vehicle or crossing the BBB could induce toxicity or unintended immune responses in neural tissues, leading to neurological side effects or exacerbating the tumor symptoms (192). Repeated use of specific delivery systems, particularly viral vectors, may lead to immune desensitization, reducing the efficacy of subsequent treatments (193). Each new delivery system must undergo rigorous safety and efficacy testing to meet regulatory standards, which is especially critical in brain tumor treatments due to the sensitive nature of the target organ (194). Lastly, the effectiveness of a delivery system can vary significantly based on the patient’s pathology, the type of tumor, its location, and the presence of metastases, necessitating adjustments or entirely different strategies (195).

Another meaningful approach, particularly relevant for central nervous system tumors, is intracranial delivery via an Ommaya reservoir, a surgically implanted device that enables the direct administration of therapeutic agents into the ventricular system or tumor cavity, thereby bypassing the blood–brain barrier entirely. It enables repeat dosing with minimal systemic exposure and consistent drug concentrations in cerebrospinal fluid. However, it also carries risks such as infection, catheter malposition, and neurotoxicity, especially if dose adjustments are not made. Despite these limitations, Ommaya-based delivery remains a valuable strategy for targeting tumors in surgically inaccessible or high-risk brain regions (196, 197).

In conclusion, selecting the optimal delivery system for brain tumor vaccines is a delicate balance between maximizing efficacy and minimizing risk. The ideal system should ensure that the therapeutic agents are delivered effectively to the tumor site while being minimally invasive and avoiding significant side effects. As research progresses, more sophisticated and safer delivery technologies are expected to emerge, potentially improving the prognosis for patients with brain tumors (198).

A promising example of this emerging experimental approach is the use of EVs. Secreted by cancer cells, copious amounts of EVs were shown to be carrying cancer-specific antigens (e.g., EGFRvIII (172),) and adjuvants (e.g., PD-L1 (170),). EVs reflect an evolving milieu of host cells and are efficacious delivery vehicles; therefore, using them in personalized vaccination approaches seems a logical next step in cancer vaccination efforts (199).

Personalized antitumor vaccines for glioblastoma involve a detailed and regulated production process, spanning from the collection of tumor samples to the administration of the vaccine. This personalization is essential due to the unique antigenic landscape of each patient’s tumor, requiring customized vaccine solutions to target individual tumor profiles effectively. As these personalized vaccines evolve, their manufacturing processes and clinical protocols vary significantly across different research groups and companies, all under strict regulatory oversight to ensure safety and efficacy (49, 60, 119).

Considering glioblastoma’s ability to evade the immune system, therapeutic vaccines aim to reinvigorate immune defenses against these established tumors, providing potential for significant, enduring antitumor effects with the latest advances in vaccine delivery technologies and a deeper understanding of T-cell memory mechanisms.

### 6.4 Regulatory hurdles/clinical implementation

Regulatory agencies set a high bar for granting approval of novel vaccines, whether they are antimicrobial or anticancer. Robust preclinical data, involving extensive animal studies, are required to satisfy regulatory agencies’ requirements, determine vaccine formulation, toxicity profiles, and potential adverse effects, and demonstrate safety and efficacy before clinical trials (200, 201). These agencies also require evidence of the vaccine’s mechanism of action and its capacity to elicit a specific and enduring immune response against cancer cells. Preventive vaccines, administered to healthy individuals, face even higher scrutiny for safety and efficacy, with potential side effects undergoing more rigorous evaluation than therapeutic interventions.

Designing and implementing clinical trials for preventive cancer vaccines, particularly for brain tumors like glioblastoma, introduces unique challenges not as pronounced in the development of therapeutics for other types of cancer. For example, the BBB poses a significant obstacle, affecting the delivery and efficacy of vaccines targeting CNS malignancies. This barrier often necessitates the development of novel delivery systems that can effectively breach or bypass the BBB to deliver therapeutic agents directly to tumor sites within the brain. However, these delivery approaches are experimental and often far from agencies’ approvals (132, 139, 140, 142, 145, 148, 154, 202, 203).

Furthermore, brain tumors such as glioblastoma are often characterized by high intratumoral heterogeneity, which complicates the identification of universal targets for vaccine development. This heterogeneity necessitates the development of personalized vaccines that target specific tumor antigens, resulting in additional regulatory complexities surrounding the customization of treatment (6, 190).

Agencies such as the FDA and the European Medicines Agency impose stringent guidelines for trial protocols, patient eligibility, and endpoints, necessitating meticulous planning and adherence to standards across all phases of clinical development (204). Regulatory agencies also mandate comprehensive data on manufacturing processes and quality control measures. Ensuring consistency, purity, and potency of vaccine formulations is crucial for meeting standards and gaining approval for commercialization. Manufacturers must comply with good manufacturing practices and undergo rigorous inspections (205).

In comparison, therapeutic interventions for non-CNS cancers often follow more established, albeit less stringent, regulatory pathways, with fewer complications related to drug delivery and a broader understanding of target antigens. This discrepancy underscores the need for specialized regulatory frameworks that accommodate the unique challenges of CNS vaccine development.

Post-marketing surveillance and pharmacovigilance are required to monitor vaccine safety and effectiveness after approval, involving the collection and analysis of real-world data on vaccine usage, adverse events, and long-term outcomes. These trials, which require lengthy durations and large participant numbers, are both costly and complex. The stringent regulatory pathway for preventive vaccines requires extensive documentation and data, often resulting in delays in market availability (18), which is particularly true for brain tumor vaccines, where the need for long-term efficacy and safety data can significantly extend the timeline for clinical development and approval.

## 7 Future directions in anti-cancer vaccine development

### 7.1 Universal vaccines or personalized therapies

The domain of cancer therapies, including those targeting brain cancers, is at a pivotal crossroads, with AI and advanced biotechnology significantly shaping the nascent treatment paradigms (20). The apparent dynamic tension between the quest to develop universal cancer vaccines and the advancement of personalized therapies highlights the role of AI in transforming research into clinical applications. Antigen discovery is fundamental to both vaccine strategies, universal and personalized, and leveraging computational, genomic, transcriptomic, and proteomic tools has exponentially expanded the pool of tumor-specific (TSA) and shared antigens (20, 123). Among them, novel sources arose from non-coding RNA (ncRNA) or epigenetic modifications in cancers (15, 206, 207), further enriching the assemblage of potential targets. Although such novel antigens are in the early stage of categorization and conceptualization, they seem particularly crucial for glioblastoma, given its low mutational load and limited antigenic epitopes that hinder effective immune responses. The discovery that many MHC-bound peptides come from ncRNAs suggests they play a crucial, if underappreciated, role in tumor immune evasion and regulation (15, 207–209). Ongoing scrutiny of the interaction between long non-coding RNA and Circular RNA-derived peptides with T cells offers promising avenues for new immunotherapy and diagnostic approaches (207, 210).

Recent advancements in personalized vaccines tailored to each patient’s genetic profile offer promising strategies in cancer immunotherapy, such as the creation of patient-specific antigens (14, 20, 21, 23, 47, 49, 60, 122, 124–126). To this end, incorporating AI-enhanced high-throughput screening technologies makes establishing Cancer Antigen Platforms within reach, allowing for the efficient identification of TSAs and TAAs while simultaneously predicting their immunogenic potential, which is poised to streamline clinical trials. For example, an established immunogenomics pipeline called the O.R.A.N. effectively identifies immunogenic antigens that are highly likely to become therapeutic targets (47). Complementarily, a TOFU platform (Tumor Open Reading Frames that are Unique), which utilizes *in vitro* transcribed mRNA technology to encode multiple tumor antigens within a single mRNA vaccine, allows the customization of virtually limitless quantities of antigens specific to each tumor type (128). In addition to the aforementioned limited availability of targetable neoantigens, developing and implementing a carrier for RNA molecules is imperative. Ben-Akiva et al. developed a class of bioreducible nanocarriers that utilize lipophilic poly(beta-amino ester) to encapsulate antigen-encoding mRNA and Toll-like receptor agonist adjuvants for the treatment of murine melanoma and colon adenocarcinoma. As mRNA encapsulation capacity is sequence-independent, this platform can also be extended to address other types of cancer.

Identifying the immunopeptidome, a diverse set of peptides presented by MHC molecules and a critical component of immune recognition and response, is the first indispensable step in creating a vaccine. In the case of glioblastoma, where biopsy material can be used to select and culture GSCs (212) efficiently, the source of the MHC-bound peptidome may include a diverse array of neoantigens originating from various organelles, such as the nucleus, mitochondria, or EVs (181). While the proteasome has been viewed as the primary source of peptides for MHC class I molecules, inhibition of the proteasome does not uniformly reduce MHC peptide presentation (211, 212). In some cases, the presentation of specific peptides even increased, suggesting the existence of alternative proteolytic pathways, with those involving autophagosomes, lysosomes, or exosomes hypothesized as culprits (213). Combining MHC-decorated immunopeptidomes with adjuvants and delivery vehicles further complicates the process. Therefore, exploring naturally formed MHC complexes on the surface of EVs that can simultaneously serve as antigen-carrying vehicles (e.g., PD-L1 (170);) may provide a valuable delivery tool for bioactive molecules that support antitumor immunity by presenting tumor antigens to immune cells. For example, DCs loaded with tumor-derived exosomes have been used in clinical trials to stimulate immune responses in patients with advanced non-small cell lung cancer, demonstrating a novel therapeutic approach that leverages the natural antigen-presenting properties of these vesicles (214, 215). With neoantigen prediction supported by AI algorithms, analyzing genetic data to predict neoantigens unique to each patient will optimize vaccine design and reduce development times.

Integrating mass spectrometry pipelines and AI technologies in immunopeptidome research revolutionizes the identification and characterization of ncRNA-derived neoantigens. By employing advanced techniques such as LC-MS/MS, Ribo-Seq, and integrative proteogenomics, researchers uncover a fertile landscape of immunogenic peptides that can be harnessed for personalized cancer therapies (20, 123). Targeted MS techniques, such as parallel reaction monitoring (PRM) and selected reaction monitoring, are also essential for accurately detecting low-abundance peptides (216). To ensure reliability, validating non-canonical peptides requires corroboration of MS findings with experimental methods.

Robust experimental therapeutic strategies utilize oncoOVs that directly lyse cancer cells while modulating immune responses within the TME (173). The dynamic nature of antigen presentation during such therapeutic interventions diversifies the peptide landscape on MHC molecules, including peptides generated through stress-related reprogramming of cellular translation and RNA processing (217). Cellular responses to the damage and stress inflicted by cytotoxic therapies, such as chemotherapy or radiation, affect the translation of RNAs into bioactive peptides. Such therapeutic stress can thus modulate the spectrum of neoantigens (122), unveiling novel targets for immunotherapy and enhancing antitumor responses.

Formulating vaccines with effective adjuvant-antigen interactions requires extensive testing and optimization. The regulatory and safety hurdles for novel and complex adjuvant formulations require substantial scrutiny, especially when targeting recurrent or metastatic brain tumors. To translate pioneering research into viable clinical treatments, startups that incorporate an AI-supported pipeline, including patient-specific antigen profiling, a vaccine formulation bench, and a vaccine activity prognostic test (e.g., a phase zero clinical trial of ex vivo PBMC activity), are necessary. They would enhance collaboration between researchers and tech developers, thereby speeding the commercialization of innovative vaccines and therapies and addressing the commercialization challenges that often slow down clinical deployment. Additionally, as with other emerging therapies, Unified Platforms for Seamless Care combines personalized immunotherapy with digital health tools crucial for enhancing patient outcomes. The future of anticancer therapy, particularly in brain cancer treatment, is moving towards a fusion of universal and personalized approaches. The field aims to enhance therapeutic efficacy and tailor treatments to individual patient profiles by harnessing AI and novel biotechnological advances, heralding a new era of precision oncology. This evolution is driven by the synergy of science, technology, and innovative digital health solutions, setting the stage for significant advancements in the fight against cancer.
